# Developing Innovative Apolar Gels Based on Cellulose Derivatives for Cleaning Metal Artworks

**DOI:** 10.3390/gels10110747

**Published:** 2024-11-16

**Authors:** Andrea Macchia, Camilla Zaratti, Davide Ciogli, Giovanni Rivici, Silvia Pilati, Nereo Sbiri, Tilde de Caro, Maria Assunta Navarra

**Affiliations:** 1 YOCOCU APS, Youth in Conservation of Cultural Heritage, Via Torquato Tasso 108, 00185 Rome, Italy; info@lab4green.it (A.M.); davide.ciogli@emec.it (D.C.); ricerca@beaumontitalia.it (G.R.); laboratorio@beaumontitalia.it (S.P.); yococu.yococu@gmail.com (N.S.); 2 CNR-ISMN, Strada Provinciale 35 d n. 9, 00010 Rome, Italy; tilde.decaro@cnr.it; 3 Department of Chemistry, La Sapienza University of Rome, Piazzale Aldo Moro 5, 00185 Rome, Italy; mariassunta.navarra@uniroma1.it

**Keywords:** physical gel, HLB, innovation, sustainability, green chemistry, cleaning polychrome artworks

## Abstract

The use of organic solvents, particularly those of a non-polar nature, is a common practice during cleaning operations in the restoration of polychrome artworks and metallic artifacts. However, these solvents pose significant risks to the health of operators and the environment. This study explores the formulation of innovative gels based on non-polar solvents and cellulose derivatives, proposing a safe and effective method for cleaning metallic artworks. The study is focused on a toxic apolar solvent, Ligroin, identified as one of the most widely used solvents in the cultural heritage treatments, and some “green” alternatives such as Methyl Myristate and Isopropyl Palmitate. The main challenge lies in overcoming the chemical incompatibility between non-polar solvents and polar thickening agents like cellulose ethers. To address this problem, the research was based on a hydrophilic–lipophilic balance (HLB) system and Hansen solubility parameters (HSPs) to select appropriate surfactants, ensuring the stability and effectiveness of the formulated gels. Stability, viscosity, and solvent release capacity of gels were analyzed using Static Light Multiple Scattering (Turbiscan), viscometry, and thermogravimetric analysis (TGA). The efficacy of cleaning in comparison with Ligroin liquid was evaluated on a metal specimen treated with various apolar protective coatings used commonly in the restoration of metallic artifacts, such as microcrystalline waxes (Reswax, Soter), acrylic resins (Paraloid B44), and protective varnishes (Incral, Regalrez). Multispectral analysis, digital optical microscopy, FTIR spectroscopy, and spectrocolorimetry allowed for the assessment of the gels’ ability to remove the different protective coatings, the degree of cleaning achieved, and the presence of any residues. The results obtained highlight the ability of the formulated gels to effectively remove protective coatings from metallic artifacts. Cetyl Alcohol proved to be the most versatile surfactant to realize a stable and efficient gel. The gels based on Methyl Myristate and Isopropyl Palmitate showed promising results as “green” alternatives to Ligroin, although in some cases, they exhibited less selectivity in the removal of protective coatings.

## 1. Introduction

Hydrocarbons are commonly used solvents across various industries, including cultural heritage restoration, particularly for the removal of non-polar varnishes or as solvents for retouching paints during restoration treatments. Their high volatility, which corresponds to low diffusion into the substrate, makes these solvents ideal for cleaning applications. However, they pose significant environmental and health risks, requiring specialized protective equipment for safe use. To investigate which toxic solvent is most commonly used in the field, this study began with a survey conducted by the YOCOCU APS association, in which thirty-two restorers with varying levels of experience participated. The survey revealed that among non-polar and toxic solvents, the most frequently used is Ligroin.

Ongoing research aims to identify more eco-friendly alternatives to these solvents [1,2]. Recently, the use of FAMEs (Fatty Acid Methyl Esters) has been explored as a potential substitute. These alternatives, however, typically exhibit low or no volatility, which necessitates their application as gels when used in cultural heritage conservation. This approach controls the rate of solvent penetration, helping to regulate the cleaning process [3,4].

Gels effectively control solvent evaporation, limit penetration into the artwork through capillary action, and provide more precise control over the treatment application [4]. Structurally, a gel consists of a small number of inorganic particles or organic macromolecules, primarily entangled polymers, which interpenetrate a relatively large volume of liquid. This unique composition gives gels more solid-like than liquid-like characteristics. The liquid prevents the polymer network from collapsing into a compact mass, while the network restricts the liquid from flowing away. Thus, gels are often seen as an intermediate state of matter between a solid and a liquid [5,6].

Additionally, synthetic polymers, such as PVA and PMMA, can also be used [7]. In the field of art conservation, gel systems are categorized into physical gels, first introduced by Wolbers in the 1990s [8], and innovative nanostructured or gel-like systems, including responsive gels and peelable systems, as outlined by Gueidão et al. [9]. Zhang et al. [10] further classified gels into several types, including supramolecular gels, metal–organic gels, dynamic covalent gels, polymer gels, inorganic gels, and organogels. Organogels are three-dimensional networks of physical gels where an organic solvent is dispersed within a structuring agent. Like other types of gels, organogels can be of both natural and synthetic origin [11].

In the context of cleaning polychrome artworks, gels offer an effective and less invasive approach, allowing conservators to better preserve the integrity of the artwork. Restorers frequently prefer the use of physical gels [12,13]. Physical gels are furthermore divided into two main categories: strong physical gels (e.g., agar, gelatin, gellan gum), which form a semi-rigid, peelable film, and weak physical gels (e.g., xanthan gum, cellulose ethers, polyacrylics), which only form a viscous paste [14]. Cellulose derivatives are commonly used by conservators to form physical gels, as they offer high compatibility with various substrates. Their ease of preparation, whether hot or cold, is a notable advantage [15]. However, their weak mechanical properties, which result in adhesive-like behavior, can be a drawback [16].

Cellulosic materials are highly sensitive to water; it readily causes them to swell and disrupts their internal structure. For this reason, cellulose-based gels work effectively as thickeners for polar solvents and some medium- to high-polarity solvents but are unsuitable for thickening non-polar solvents, such as hydrocarbons. As a result, conservators and restorers often turn to alternative gelling agents for non-polar solvents, most commonly physical gels based on polyacrylates, such as Carbopol.

These polyacrylate gels offer stability and effective viscosity control across a broader range of solvent polarities. However, they can be challenging to prepare, and there are risks for the substrate due to the residual alkalinity from their acid–base reaction. Additionally, the gel structure itself may not always meet the ideal requirements for certain delicate restoration applications [17,18].

The aim of this study was to define a non-polar–polar emulsion using surfactants to formulate cellulose-ether-based physical organogels for cleaning polychrome artworks. 

The different compounds of the non-polar–polar system were defined and selected based on the hydrophilic–lipophilic balance value (HLB), an empirical parameter that indicates the balance between the hydrophilic and lipophile moieties of the surfactant molecule. Moreover, the HLB was compared to the Hansen solubility parameters (HSPs) and their Relative Energy Difference (RED) was calculated to evaluate the chemical affinity between the different compounds [19,20]. The HLB value also estimates the tendency of the surfactant to stabilize emulsions of non-polar substances (typically oils) in polar substances (usually water) or vice versa. A high HLB value (>10) suggests that the surfactant is more hydrophilic, and suitable for oil-in-water (O/W) emulsions, while a low HLB value (<10) indicates that it is more lipophilic, making it suitable for water-in-oil (W/O) emulsions. HSP can be used in combination with HLB to predict the miscibility and stability of emulsions. If the HSP components of the surfactant are similar to those of the non-polar or polar substance in the mixture, then the surfactant will be effective in stabilizing the system, which can then be gelled using a thickener [21].

The formation of stable microemulsions often requires high concentrations of surfactants. However, this can sometimes be reduced by using co-solvents, such as alcohols, which play a critical role in adjusting the system’s polarity, hydration, and surface activity. These co-solvents aid in forming microemulsions by optimizing the behavior of surfactants [22,23]. Co-surfactants, typically short-chain amines or alcohols, help lower interfacial tension and adjust the curvature of reverse micelles, enhancing both the solubility and stability of non-polar compounds. The choice of co-surfactant significantly impacts the overall performance of the system, affecting microemulsion properties across various applications [24]. In cosmetic formulations, alkanediols are used as co-solvents, with chain lengths ranging from C-4 to C-10 (e.g., 1-butanol, 1-pentanol, 1-hexanol) [25].

Among the different diols, 1,2-hexanediol is widely utilized, particularly in “greener” formulations, due to its eco-friendly, non-toxic properties [26]. Moreover, 1,6-hexanediol is less polar than 1,5-pentanediol because of its longer carbon chain. This reduced polarity allows hexanediol to better interact with both polar and non-polar components in emulsions, enhancing stability and promoting improved dispersion of non-polar substances [27,28].

The use of HLB values in combination with chemical affinity and emulsion scanning helped in formulating stable, easy-to-prepare apolar cellulose-ether-based gels. These gels were then applied for the cleaning of non-polar substances commonly used in the field of cultural heritage conservation. The rheology of the gels was studied to better understand solvent release and stability, as well as to evaluate their potential application in the conservation sector. The innovation of the gels presented in this study lies in the use of cellulose ethers as gelling agents of apolar solvents used in the cleaning of metal cultural goods. Cellulose ethers are widely used today in the cultural heritage sector as thickening agents but are mainly used to thicken water-based solutions or solvents with medium to high polarity. In this study, the possibility of using these cellulose derivatives to gel apolar systems is investigated and demonstrated.

## 2. Results and Discussion

### 2.1. Selection of Reference Solvents and Green Alternative

**Figure 1:**
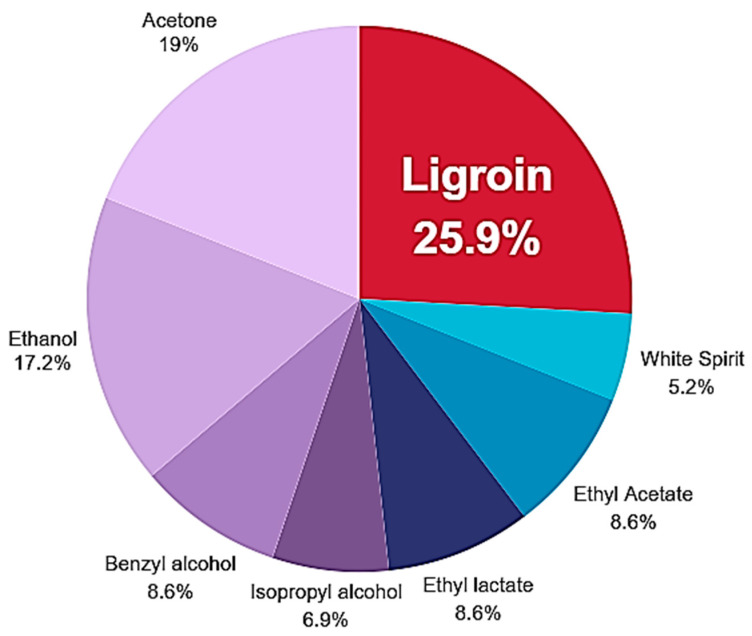
Pie chart of the most common solvent used in cleaning practices nowadays by restorers.

Figure 1 shows the percentage use of the most commonly employed solvents in the cleaning of cultural heritage materials, which, according to respondents, have an impact on both the environment and operators. Ligroin emerged as the most commonly used organic solvent. This solvent is a non-polar mixture of aliphatic hydrocarbons (petroleum ether) with an aromatic content of less than 0.5%, obtained through fractional distillation at a boiling range of 100–140 °C. It is widely used as a diluent for other solvents and serves as a solvent for Plexisol P550, acrylic adhesive 375, ketonic resins (Laropal K80), and microcrystalline waxes. Ligroin is also employed as a “washing” solvent for other solvents after solubility tests, and it is used to prepare mixtures with precise polarity for cleaning operations on polychrome surfaces.

Defining the apolar phase of the emulsion (Ligroin) for gelling and its green alternatives, the study was focused on determining the polar phase of the emulsion and identifying the most suitable surfactant to achieve a stable gel using the RED calculation.

### 2.2. Definition of Polar Phase of Emulsion

Table 1 reports the main cellulose ethers used as thickeners. To simplify the study, we analyzed the possibility of using only one cellulose ether that would still be chemically similar to the others. This affinity was assessed using the RED calculation. The different analyses conducted identified Hydroxypropyl Cellulose (Klucel-G) as a representative cellulose ether, with a RED greater than 1 only for cellulose ethers characterized by the presence of highly non-polar groups (high δD or δP values, where δD (MPa) is the contribution of the dispersion intermolecular forces, δP (MPa) is the contribution of the dipole intermolecular forces and δH (MPa) is the contribution of the hydrogen-bonding intermolecular forces).

### 2.3. Definition of Surfactants

To define the surfactants that would combine the apolar and polar phases to form a stable gel, the study employed the HLB system and used the RED calculation to evaluate the affinity of potential surfactants for both the polar and apolar phases. The first step involved calculating the RED value between Ligroin and various surfactants with HLB values within the five target ranges described in Section 4, to assess the surfactants’ chemical affinity with the apolar phase. As shown in Table 2, Cetyl Alcohol and polyethylene glycol ester of stearic acid (PEG 100 Stearate) exhibited the greatest chemical affinity for Ligroin among the surfactants evaluated. This result contrasts with theoretical predictions based solely on HLB values, which suggest that surfactants with higher HLB values should exhibit greater affinity for the polar phase. In surfactants with a long carbon chain, if the hydrophilic portion is large enough, the surfactant may still have a high HLB value. This explains why some surfactants, even with a long carbon chain, have a high HLB and yet exhibit a RED value <1 when compared to Ligroin.

The chemical affinity between the different surfactants and the polar phase of the system (Klucel-G + 1,2-hexanediol) was also studied. Table 3 reports the corresponding RED values. Similar to the previously described results, the RED evaluation in this case also led to unexpected outcomes when compared to theoretical predictions based on HLB values. For the polar phase of the system, as shown in Table 3, surfactants derived from sorbitol exhibited the highest affinity, even though their low HLB values would theoretically suggest a greater affinity for the apolar phase.

The evaluation of the HLB value compared to the RED value calculated both with respect to the polar and the apolar phase of the system defined the following surfactants as the most suitable to stabilize the experimental system (Ligorin + Klucel-G +1,2-hexanediol):

- PEG 100 Stearate (HLB~18), due to its high HLB value, is suitable for the formulation of oil-in-water (O/W) emulsions. Although Ligroin is an apolar phase, the presence of Hydroxypropyl Cellulose and 1,2-hexanediol may require good compatibility with hydrophilic phases to achieve a stable dispersion.
- Cetyl Alcohol (HLB~15), which contributes to the stabilization of O/W emulsions by acting both as a surfactant and as a structuring agent. Due to its ability to reinforce the internal structure of the emulsion, it improves the viscosity and overall stability of the system.
- Tefose^®^ 63 (HLB~9.5) is a versatile surfactant produced by Gattefossè (Saint-Priest, France), capable of stabilizing both oil-in-water (O/W) and water-in-oil (W/O) emulsions. It consists of a mixture of PEG-6 (MW 300) esters of stearic (C_18_) acid and ethylene glycol esters of palmitic (C_16_) and stearic acids and PEG-32 (MW 1500) esters of stearic acid. In this case, its use allows for a balanced formulation, providing a good compromise between the hydrophilic and lipophilic phases, thereby adapting to the characteristics of Ligroin.
- Mix of Tween 20 (HLB~16) and Span 80 (HLB~4), selected to realize an O/W system by the complementary interaction between the two surfactants. Tween 20, being highly hydrophilic, stabilizes the polar phase, while Span 80, which is strongly lipophilic, reduces the interfacial tension with the apolar phase of Ligroin, improving the dispersion and stability of the emulsion.
- Span 80 and Span 60 (HLB of 4 and 4.7, respectively) are lipophilic surfactants used to stabilize water-in-oil (W/O) emulsions. Due to their low affinity for the polar phase and high affinity for the apolar phase, they ensure a stable dispersion of the polar phase within the oil phase containing Ligroin.

### 2.4. Determination of Minimum Amount of Surfactants, Optimization of the Polar Phase/Apolar Phase Ratio

After selecting the apolar solvent to be gelled, the thickening agent, and the surfactants for the study, the formulation was optimized by determining the minimum % of surfactant needed to stabilize the system. The surfactant chosen for the test was the Tween20/Span 80 mix, as it had a medium HLB value compared to the other surfactants. The test indicated that the minimum amount of surfactant required for the formulation is 10% relative to the total volume of the polar and apolar phases. The following table (Table 4) shows the turbidity measurements of the various formulated emulsions.

**Figure 2:**
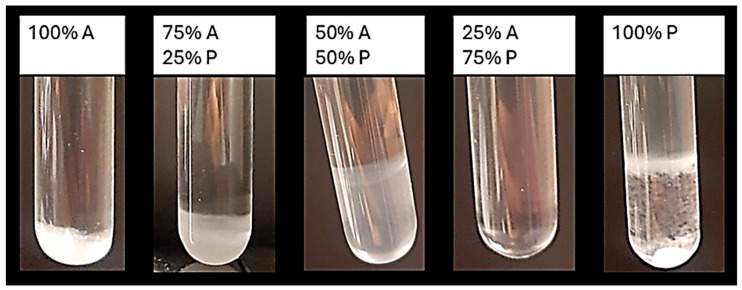
Macroscopical observations of the tubes prior to turbidity measurements. A stands for “Apolar phase” while P stands for “Polar phase”.

An additional scanning procedure based on test tubes was performed to determine the optimal percentage of the apolar phase (Ligroin) and the polar phase (Klucel-G + 1,2-Hexanediol) required to obtain a clear and stable emulsion (Figure 2). The table below (Table 5) presents the turbidity measurements of the different emulsions prepared for this scanning process. Emulsions characterized by a turbidity value of < 30 NTU and a higher percentage of dispersed apolar phase were considered suitable for the subsequent steps of the experiment. However, the use of Tefose^®^ 63 and the Tween 20/Span 80 mix did not yield a transparent gel suitable for the following cleaning tests. Based on the results obtained, the optimal component % for the different surfactants are as follows:

- Cetyl Alcohol: 50 polar phase/50 Ligroin.
- PEG 100 Stearate: 50 polar phase/50 Ligroin.
- Span 60: 25 polar phase/75 Ligroin.

### 2.5. Gel Formulation for the Cleaning Test and Definition of Rheological Properties of the Gels

The results obtained highlighted the potential to formulate stable gels that maximize the apolar phase content by using Cetyl Alcohol, PEG 100 Stearate, and Span 60 as surfactants. These surfactants were selected for the formulation of gels intended for cleaning tests on laboratory-prepared samples. Gels were prepared using Ligroin as the apolar phase, along with gels in which Ligroin was replaced by potential green alternatives: Isopropyl Palmitate and Methyl Myristate (Table 6). For all the gels, the polar phase consisted of Klucel-G (the thickening agent) and 1,2-hexanediol (the co-surfactant).

### 2.6. Stability Measurements and Viscosity Analysis

**Figure 3:**
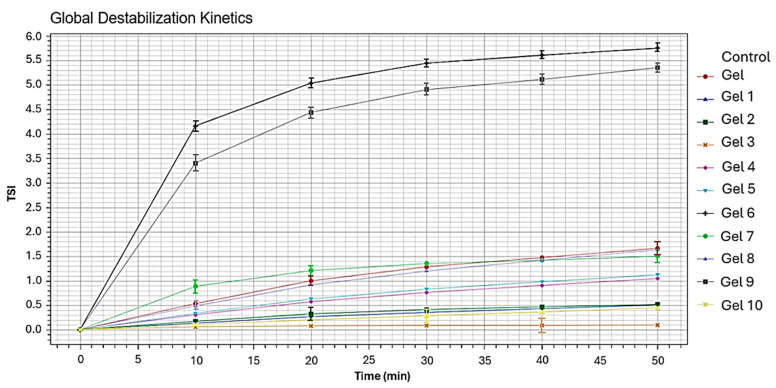
Global Destabilization Kinetics of the formulated gels.

The results of the analysis conducted with the Turbiscan are presented in Figure 3. The plot is related to the Global Destabilization Kinetics, which shows the evolution of the Turbiscan Stability Index (TSI) for the different gels over time, providing a clear and comprehensive view of the stability evolution of the analyzed systems. An increase in the TSI corresponds to an increase in the system’s instability. The Gel 6 and Gel 9 samples showed a high TSI, indicating rapid destabilization, with clear signs of instability observed within the first 10 min of measurement. Phase separation occurred in these two samples. In contrast, the gels numbered 1 to 5 and 7 to 10 recorded lower TSI values, suggesting greater structure stability compared to Gel 6 and 9. Among these, Gel 3 exhibited the lowest TSI value, indicating it is the most stable sample among those analyzed. Gels 1, 2, 4, 5, 7 and 8 showed some variability in particle distribution but demonstrated good system stability over time. Methyl Myristate and Isopropyl Palmitate were used, in combination with Span 60, to formulate Gels 6 and 9, respectively. The surfactant could be the cause of the system’s instability. Over time, an unstable structure may result from the surfactant’s polar phase interacting more with the solvent molecule’s polar portion and reducing its ability to bond with the Klucel-G. Ligroin and Span 60 were used to formulate Gel 3, which was more stable. Since the Ligroin is less polar than Isopropyl Palmitate and Methyl Myristate, in this case, there is a greater interaction between the surfactant and the Klucel-G leading to a more stable formulation over time.

Table 7 reports the viscosity of the formulated gels. This parameter is correlated with the HLB value of the surfactant. Gels formulated with higher HLB surfactants, such as PEG 100 Stearate, demonstrate higher viscosity, while those with lower HLB surfactants, like Span 60, show reduced viscosity. The increase in viscosity with higher HLB values suggests that more hydrophilic surfactants stabilize the apolar phase more effectively, leading to more rigid structures. These findings align with theoretical expectations and the existing literature, indicating that more hydrophilic surfactants contribute to improved gel stability [29,30,31].

### 2.7. Thermogravimetric Analysis (TGA)

**Figure 4:**
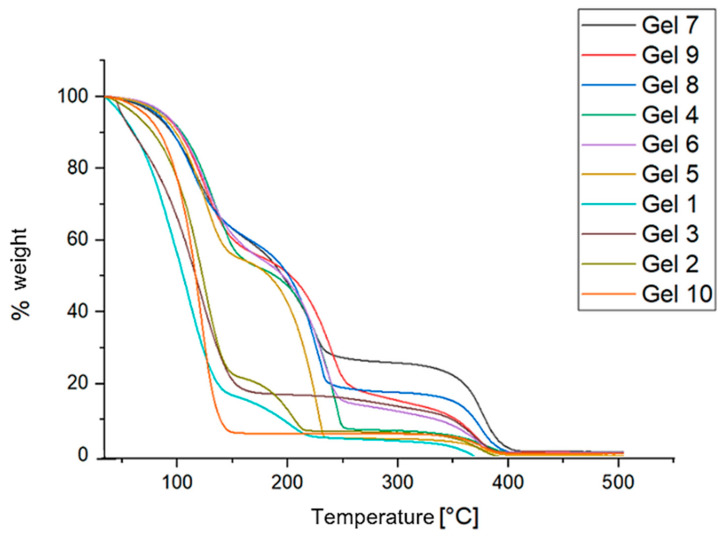
Termogravimetric curves of the different gels.

**Figure 5:**
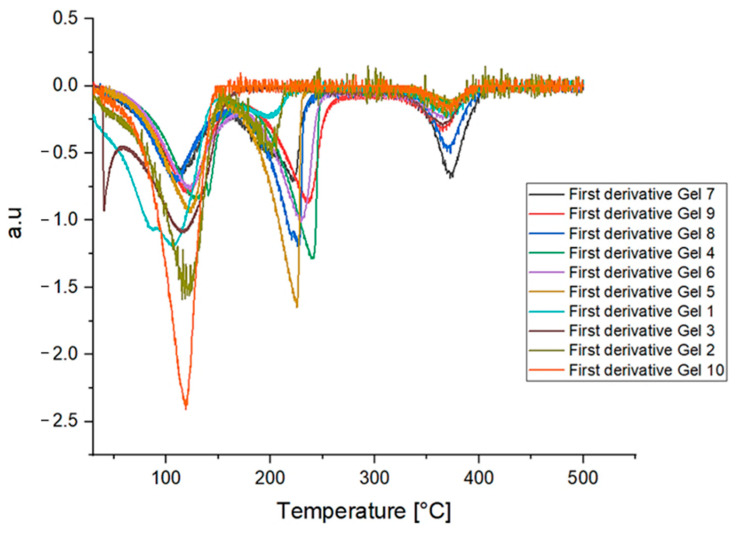
First derivative curves of the thermogravimetric curve related to all the different gels formulated.

The following figures (Figure 4 and Figure 5, respectively) present the results obtained from thermogravimetric analysis (TGA), including the TGA curves and their respective derivatives. The analysis provides insights into the thermal stability of the gels, indicating how each formulation responds to increasing temperatures and at which point significant mass loss (due to solvent evaporation) occurs.

Gels formulated with Methyl Myristate exhibited a slower mass change as a function of temperature compared to those formulated with Ligroin or Isopropyl Palmitate as the apolar solvent. This indicates better retention of materials within the system, which is influenced by the properties of Methyl Myristate itself. With its lower volatility, higher boiling point, and lower surface tension compared to Ligroin and Isopropyl Palmitate, Methyl Myristate helps the gel maintain stability over a wider temperature range.

Gel 7 (black line) and Gel 9 (red line) displayed a more gradual mass loss compared to the other gels, retaining a larger percentage of their mass as the temperature increased. Of the two, Gel 7 exhibited a higher and flatter mass change curve, indicating that it retains its components at higher temperatures more effectively than the others.

### 2.8. Cleaning Tests

The formulated gels were then applied to the copper frame, which had been previously coated with different layers of apolar protective coatings, to evaluate their effectiveness in removing these protective layers. This study aimed to replace the toxic solvents currently in use, such as Ligroin, with safer, green solvents in gel form, as well as to compare their performance to green solvents in liquid form.

### 2.9. Evaluation of Cleaning Efficacy of Gels

#### 2.9.1. UV Fluorescence

To macroscopically document the cleaning effectiveness of the different gels, images were taken under various lighting conditions: visible light, ultraviolet (UV), and filtered ultraviolet (excluding the near-UV component of the UV lamp). The evolution of the copper frame’s appearance was monitored, starting with the oxidation process. Additional images were collected after applying various protective layers and following the cleaning. No significant macroscopic changes were observed on the metal surface’s aspect after applying the protective coatings, with the exception of the Regalrez varnish, which resulted in increased color saturation and surface brightness, particularly visible on the right side of the copper frame in the pictures present in Table 8. Further images were captured after the artificial aging process to evaluate any visible alterations. Some changes in aspect are evident, particularly in the UV images (Table 8), where variations in fluorescence were observed for the application of the multilayer protective coating realized with Incral and Soter wax. This effect is likely due to the degradation of benzotriazole, a compound known for its sensitivity to moisture. The other protective coatings did not show significant fluorescence variations after the aging process.

#### 2.9.2. Optical Microscopy

The observations collected from the coated surfaces before the cleaning tests, along with the microscopic observations taken afterward, revealed significant differences in the appearance of the copper frame surface. The images acquired both before and after the cleaning tests, documented in Supplementary Materials, clearly show these changes.

In most cases, a reduction in surface brightness was noted after cleaning, which can be attributed to the removal of the protective coatings. Specifically for the Regalrez varnish, the results established an efficacy order for the gel formulations as follows:

Gel 1 ≥ Gel 2 ≥ Gel 3
Gel 5 > Gel 4 > Gel 6
Gel 7 ≥ Gel 8 > Gel 9

Regarding the Reswax coating, the cleaning efficacy order was evaluated as follows:

Gel 3 > Gel 1 > Gel 2

The change in the solvent in the gel formulation did not significantly modify the cleaning efficacy of the same surfactants. In the case of the surface treated with a multilayer application of Reswax and Paraloid B44, the cleaning treatment, performed with the formulated gels, effectively and selectively removed just the Reswax layer, leaving the underlying Paraloid B44 layer intact. Where selectivity was high, an increase in surface brightness was observed due to the removal of the wax while preserving the varnish. Gel 1 (Ligroin + PEG 100 Stearate) was not selective, removing both the Reswax and the underlying Paraloid layer. Gel 1 obtained a cleaning process that was too invasive, compromising the integrity of the protective varnish. In contrast, Gels 2 and 3 showed higher selectivity, successfully removing the outer wax layer. Unfortunately, both gels slightly solubilized the Paraloid B44 varnish layer, leading to an unintended side effect: the surface became sticky, making the gel more difficult to remove. During the cleaning process, cotton fibers from the swabs used for gel removal were observed to have become trapped in the partially dissolved Paraloid B44 layer. For the “green solvents,” the following order of cleaning effectiveness was established:

- Gel 5 (Methyl Myristate + Cetyl Alcohol) > Gel 6 (Methyl Myristate + Span 60) > Gel 4 (Methyl Myristate + PEG 100 Stearate).
- Gel 7 (IPP + PEG 100 Stearate) > Gel 8 (IPP + Cetyl Alcohol) > Gel 9 (IPP + Span 60).

Overall, for this type of multilayer protective coating, the results identified the following order of cleaning efficacy:

- Gel 2 (Ligroin + Cetyl Alcohol) > Gel 3 (Ligroin + Span 60) > Gel 1 (Ligroin + PEG 100 Stearate).

Regarding the Zapon coating, it was not possible to appreciate changes in the surface after the cleaning treatment. In the case of the surface treated with a multilayer of Incral varnish + Soter wax, as was previously observed in the application of Paraloid B44 + Reswax, the cleaning treatment performed using the formulated gels led to the selective removal of the Soter wax, leaving the underlying Incral varnish layer unchanged or partially unchanged. The gels were nevertheless more selective compared to using liquid Ligroin, which solubilized both protective layers. The cleaning efficacy of the gels was ranked as follows:

- Gel 2 (Ligroin + Cetyl Alcohol) > Gel 3 (Ligroin + Span 60) > Gel 1 (Ligroin + PEG 100 Stearate).
- Gel 5 (Methyl Myristate + Cetyl Alcohol) > Gel 4 (Methyl Myristate + PEG 100 Stearate) > Gel 6 (Methyl Myristate + Span 60).
- Gel 7 (IPP + PEG 100 Stearate) > Gel 9 (IPP + Span 60) > Gel 8 (IPP + Cetyl Alcohol).

Lastly, regarding the Soter wax, the considerations previously made for the application of Reswax and Zapon also apply here. The cleaning efficacy of the gels was ranked as follows:

- Gel 3 > Gel 1 > Gel 2.
- Gel 5 > Gel 4 > Gel 6.
- Gel 9 > Gel 8 > Gel 7.

#### 2.9.3. Spectrocolorimetry

To assess the surface changes induced by the cleaning, total color variation (ΔE) was used by calculating between the original surface, prior to the application of the protective coatings, and the surface after the cleaning treatments.

Table 9 reports the results obtained for the various gels tested. The following observations can be made from the data:

- Regalrez varnish: Minimal color variation (ΔE = 1.3 for Gel 6) observed for all gels, except for Gel 9 (ΔE = 7.2).
- Reswax: Minimal color variation for all gels (ΔE = 2 for Gel 8), except for Gel 4 and Gel 3.
- Reswax + Paraloid B44: Minimal color variation for all gels (ΔE = 1.7 for Gel 4), except for Gel 2 and Gel 3 (ΔE = 6.7 and 5, respectively).
- Zapon: Minimal color variation for all gels (ΔE < 1.7 for Gel 5 and 6), except for Gel 9 and Gel 4 (ΔE = 8 and 5.9, respectively).
- Soter wax + Incral varnish: Minimal color variation for all gels, except for Gel 10, Gel 8, and Gel 7 (ΔE = 4.8, 4.9 and 6, respectively).
- Soter wax: No detectable color change for all gels tested.

Table 9 shows the ΔE values calculated for the different gels applied to each protective coating.

#### 2.9.4. Fourier Transform Infrared Spectroscopy (FTIR) in Attenuated Total Reflectance (ATR) Mode

To assess the potential presence of gel and/or solvent residues on the metallic surface of the copper frame following the cleaning process, spectra were collected at multiple stages: before and after the application of the different protective coatings, after the artificial aging cycle, and finally post-cleaning.

**Figure 6:**
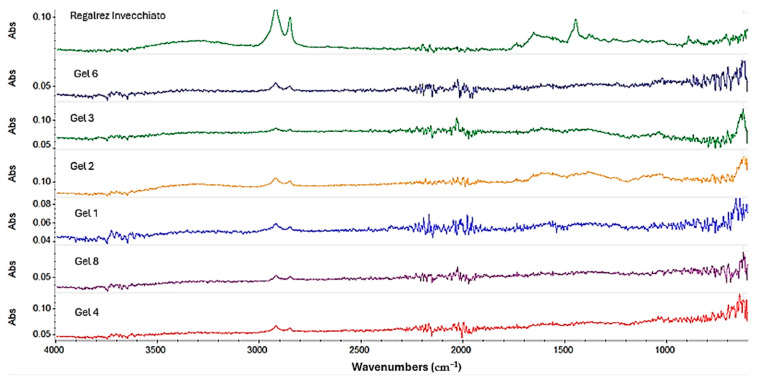
Spectra acquired on the copper frame treated with Regalrez varnish.

For the Regalrez varnish, the gels formulated with Ligroin and the three different surfactants (Figure 6) all effectively removed the Regalrez resin-based protective coating. However, the collected spectra still revealed a slight presence of the protective layer, indicated by residual aliphatic groups at 2915 cm^−1^ and 2848 cm^−1^. Among these, the gel formulated with Ligroin + Span 60 (Gel 3) demonstrated the best cleaning result.

For the gels formulated with ‘green solvents’, while the removal of the protective coating was generally good, it was not entirely optimal. Peaks in the spectral region between 3000 and 2500 cm^−1^, indicative of remaining aliphatic compounds, were still present. Of these, the gel formulated with Isopropyl Palmitate yielded the most favorable cleaning outcome.

**Figure 7:**
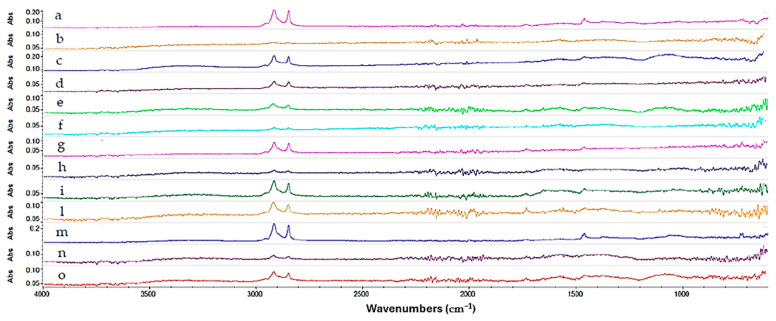
Spectra acquired on the copper frame treated with Reswax protective coating. In magenta the Reswax spectrum (a), in orange the liquid Ligroin (b), in blue Gel 1 (c), in purple Gel 3 (d), in green Gel 2 (e), in turquoise Methyl Myristate liquid (f), in light purple Gel 6 (g), in blue Gel 4 (h), in dark green Isopropyl Palmitate liquid (i), in dark yellow Gel 7 (l), in light blue Gel 5 (m), in violet Gel 9 (n) and in red Gel 8 (o).

Regarding the Reswax coating, for the gels formulated with Ligroin as the solvent, although the gels were effective in removing the coating, some peaks related to Reswax are still present, indicating that the removal was not uniform. The bands at 2915 cm^−1^ and 2848 cm^−1^ can be associated with the stretching of the C-H bond of the aliphatic wax chains. The weak band at 1730–1710 cm^−1^ is related to the stretching of the C=O carbonyl bond. The bands present in the region of the spectrum between 1500 cm^−1^ and 1300 cm^−1^ (1472, 1461, 1376 cm^−1^) are associated with the C-H and C-O bond stretching. Gel 1 and Gel 3 were the most efficient in removing the protective coating, as highlighted in Figure 7 by the reduction in the intensity of the absorption peaks related to the coating. In the case of Gel 4, a significant reduction in the peaks related to the C-H bond is also observed, suggesting that the gel formulated using this surfactant (PEG 100 Stearate) was the most effective solution for removing the Reswax layer. The following image (Figure 7) shows a comparison of all the spectra acquired for the different gels tested on the Reswax coating.

**Figure 8:**
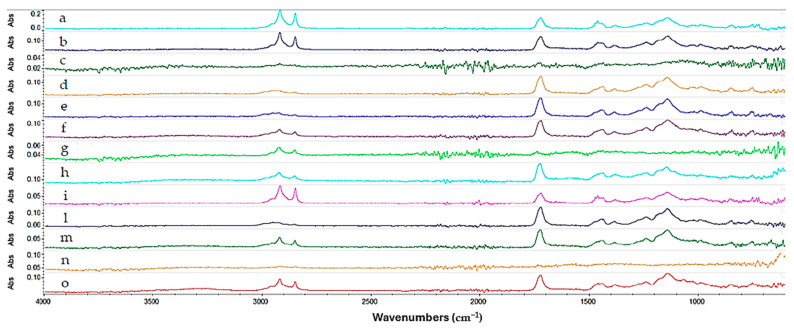
Spectra acquired on the copper frame treated with Reswax + Paraloid B44 multilayer protective coating. Starting from the top: in turquoise not aged Reswax + Paraloid B44 spectrum (a), blue aged Reswax + Paraloid B44 spectrum (b), in dark green Gel 7 (c), in dark yellow Isopropyl Palmitate liquid (d), in blue Methyl Myristate liquid (e), in purple Gel 4 (f), in light green Gel 5 (g), in cerulean Gel 8 (h), in magenta Gel 9 (i), in dark blue liquid Ligroin (l), in green Gel 3 (m), in orange Gel 1 (n), and in red Gel 2 (o).

Regarding the multilayer coating composed of Reswax and Paraloid B44 (Figure 8), the spectrum shows (turquoise line) a strong absorption between 3000 cm^−1^ and 2800 cm^−1^, attributable to the stretching of the aliphatic C-H bond indicating the presence of the waxy layer of Reswax applied on top of the Paraloid B44 layer. From the spectrum, peaks at 1722 cm^−1^ (C=O), 1462, 1380, 1235, 1139, 1023, 989, and 750 cm^−1^ are indicative of the presence of Paraloid B44 resin. In this case, good results were obtained with Gels 4, 3, 7, 8, and 9. They removed the protective layer, but not uniformly. Gels 1 and 2 were more effective, but they removed both protective coatings, thereby failing to maintain good selectivity. All the gels that were able to selectively remove the wax while leaving the underlying Paraloid B44 intact still led to its partial solubilization, making the surface sticky and making gel removal more challenging. The image below (Figure 8) shows the spectra collected.

**Figure 9:**
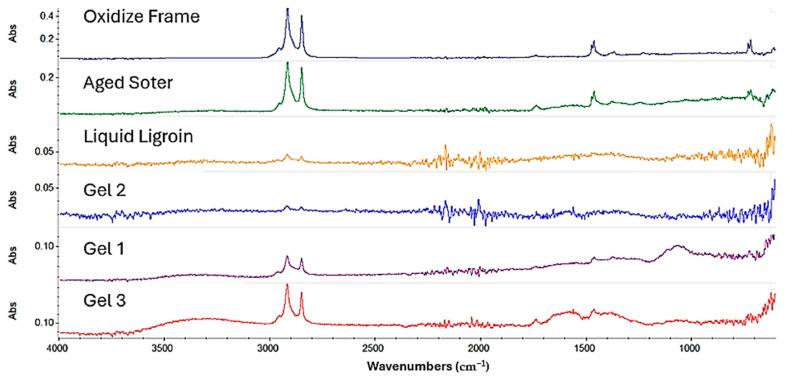
Spectra acquired on the copper frame treated with Soter protective coating treated with the gels formulated with Ligroin solvent.

Regarding the Soter protective coating, among the gels formulated with Ligroin, Gel 2 gave the best result in terms of removal of this particular coating. For the gels formulated with Methyl Myristate as the apolar solvent, the best result was obtained with Gel 6, although good results were also achieved with Gel 4. Concerning the gels formulated with Isopropyl Palmitate as the solvent, the best results were obtained with Gel 8, while the other formulations were not able to successfully remove the Soter coating. Absorption bands in the region 2850–2950 cm^−1^ are related to the C-H and C-H_2_ aliphatic stretching groups. Figure 9 shows the comparison between the spectra collected.

**Figure 10:**
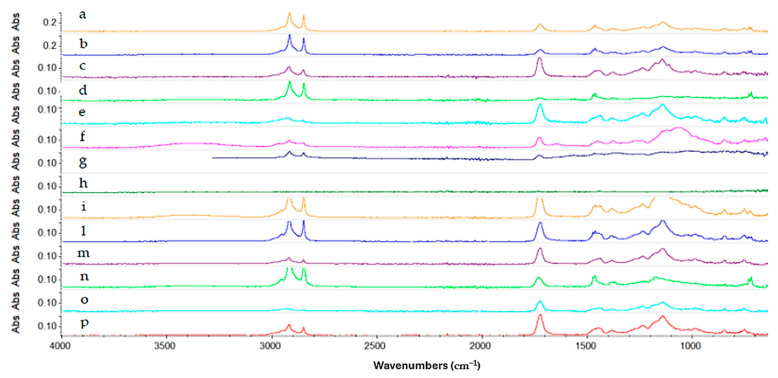
Spectra acquired on the copper frame treated with Soter wax + Incral varnish multilayer protective coating after the gel cleaning test. Starting from the top: in dark yellow the multilayer protective coating spectrum (a), in blue the multilayer coating aged (b), in purple liquid Isopropyl Palmitate (c), in light green Gel 9 (d), in turquoise Gel 8 (e), in magenta Gel 7 (f), in dark blue Gel 5 (g), in dark green Gel 1 (h), in orange Gel 3 (i), in light blue liquid Ligroin (l), in light purple Gel 1 (m), in greenish Gel 6 (n), in turquoise liquid Methyl Myristate (o), and in red Gel 4 (p).

Regarding the Soter and Incral varnish multilayer coating, among the gels formulated with Ligroin as the solvent, only Gel 1 was able to effectively remove the protective coating. However, it removed both layers, failing to maintain a good level of selectivity. In the spectra, the absorption bands visible in the region between 2850 and 2950 cm^−1^ are related to the present of the Soter wax applied over the Incral varnish. While the absorption bands at 1722 cm^−1^ (C=O), 1462, 1380, 1235, 1139, 1023, 989, and 750 cm^−1^ are indicative of the presence of Incral varnish. For the Methyl Myristate gels, better results were obtained with Gel 5, which removed the coating, though not uniformly. Similar results were obtained with the Isopropyl Palmitate gels. Figure 10 shows the spectra acquired for this multilayer protective coating.

**Figure 11:**
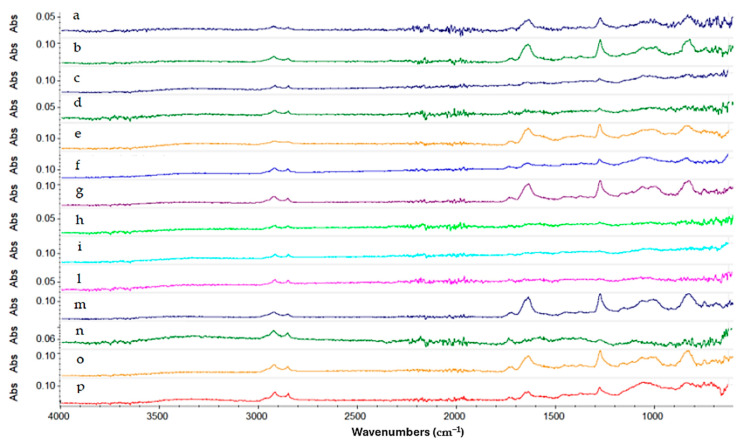
Spectra acquired on the copper frame treated with Zapon protective coating after the gel cleaning test. Starting from the top: in dark blue the not aged Zapon spectrum (a), in green aged Zapon spectrum (b), in purple liquid Ligroin (c), in dark green Gel 3 (d), in dark yellow Gel 1 (e), in light blue Gel 2 (f), in purple liquid Methyl Myristate (g), in light green Gel 5 (h), in turquoise Gel 4 (i), in magenta Gel 8 (l), in dark blue Gel 6 (m), in dark green Gel 7 (n), in orange Gel 9 (o), and in red liquid Isopropyl Palmitate (p).

Lastly, for the Zapon coating, Gel 2 and Gel 3 gave good results, while Gels 4, 5, and 6 were all able to remove the coating effectively. Regarding the gels formulated with Isopropyl Palmitate, both Gel 8 and Gel 7 achieved a high level of cleaning efficacy. However, Gel 6 was not able to remove the coating, as highlighted by the FTIR spectrum, where the absorption bands related to the Zapon are still visible. Figure 11 shows the comparison between all the spectra acquired for this coating.

The selection of the components for the apolar phase was carried out by calculating the RED using the HSP of various cellulose ethers. This analysis led to the choice of Klucel-G as the thickener due to its high chemical affinity with other potential thickeners commonly used in the field. The selection of hexanediol as a co-surfactant was based on data from the scientific literature.

The selection of surfactants for the gel formulations was made using a combined approach that considered both HLB value and HSP. This allowed for a targeted selection of surfactants that ensured compatibility with the proportions of the apolar and polar phases in the system, promoting stable emulsions. The HLB calculation, performed for different percentages of Ligroin, identified four target values (4, 8, 12, and 16), and the nonionic surfactants were chosen accordingly. One surfactant with a higher HLB value was also tested due to its high hydrophilicity, which could stabilize systems with a significant polar phase.

The use of HSP and RED further refined the selection by comparing the chemical affinity between the surfactants and the other components of the gel. This method helped to ensure compatibility between the apolar phase (ligorin) and the polar phase for long-term stability. The result of the RED calculation selection involved five surfactants with different HLB values: Cetyl Alcohol, Tefose 63, PEG 100 Stearate, a mix of Tween 20 and Span 80, and Span 60. It was noted that while the HLB parameter is helpful for emulsion formulation, it alone is insufficient for selecting the optimal surfactant.

In determining the minimum surfactant concentration required for stabilizing the emulsions, test tube experiments revealed that a concentration of 10% of the total volume was optimal for achieving a stable emulsion. Turbidity analyses confirmed that this concentration effectively stabilized the apolar phase (Ligroin) in the polar phase, preventing coalescence and ensuring uniform dispersion.

The optimal formulation ratios for gel stability were as follows:

- Cetyl Alcohol: 50% polar phase/50% Ligroin.
- Span 60: 25% polar phase/75% Ligroin.
- PEG 100 Stearate: 50% polar phase/50% Ligroin (very dense).

For systems based on Isopropyl Palmitate (IPP), an apolar/polar phase ratio of 25:75 was needed, reflecting IPP’s different solubility compared to Ligroin.

Rheological analyses, using Turbiscan, indicated that the gel formulated with Ligroin and Span 60 (Gel 3) exhibited the best stability, confirming the effectiveness of Span 60 in providing the gel with a strong viscoelastic structure. Viscosity analyses showed that gel viscosity increased with higher HLB values of the surfactants used, with Cetyl Alcohol and PEG 100 Stearate delivering excellent results. This aligns with the concept that higher HLB surfactants enhance polar system stability.

Thermogravimetric analysis (TGA) revealed that Gel 7 and Gel 9 experienced more gradual mass loss, maintaining more weight at higher temperatures, suggesting a potential advantage for slow solvent release in cultural heritage applications.

Optical microscopic observations of surfaces treated with different gels demonstrated effective removal of the Regalrez protective coating by Gel 1 (Ligroin + PEG 100 Stearate) and Gel 2 (Ligroin + Cetyl Alcohol). Gels formulated with Methyl Myristate and Isopropyl Palmitate also showed good results, albeit to a lesser extent. Sprectocolorimetric analysis confirmed the successful removal of Regalrez with all gels.

The following is a summary of results for different protective coatings:

- Gel 2 (Ligroin + Cetyl Alcohol): The most versatile gel, performing well for Regalrez, Reswax, Soter, Zapon, and Reswax + Paraloid B44.
- Gel 3 (Ligroin + Span 60): Excellent for Regalrez, Soter, and Zapon, but less effective for complex combinations like Soter + Incral and Reswax + Paraloid B44.
- Isopropyl Palmitate and Methyl Myristate-based formulations (especially with Cetyl Alcohol) were effective but showed uneven or non-selective removal for certain coatings like Regalrez and Zapon.

All the gels achieved results comparable to the commercial product (Gel 10) used as a reference for the cleaning tests.

In conclusion, the optimal gel depends on the specific protective coating-substrate combination, with Cetyl Alcohol proving to be the most effective surfactant across the various protective coatings tested.

The surfactants employed in this study demonstrate distinct stabilization mechanisms based on their molecular structure, particularly their hydrocarbon chain length and polar group characteristics. Their structural features directly influence their performance in gel formation and stability.

The molecular architecture of these surfactants significantly impacts their behavior at the polar–apolar interface. Cetyl Alcohol (C_16_H_34_O), with its linear and medium-length hydrocarbon chain, achieves an optimal balance between polar and apolar interactions. Its moderate HLB value facilitates effective emulsion stabilization through balanced affinity for both phases, resulting in medium-viscosity gels with good stability characteristics. The C16 chain provides sufficient hydrophobic interactions while maintaining adequate mobility at the interface. Span 60 (C_18_H_34_O_5_) exhibits enhanced performance in terms of gel cohesion and stability, primarily due to its longer hydrocarbon chain and lower HLB value. The increased chain length promotes stronger hydrophobic interactions, leading to more effective stabilization of the apolar phase. This molecular characteristic explains the formation of highly cohesive gels with superior viscosity and stability, as confirmed by Turbiscan analyses. The pronounced lipophilic character of Span 60 makes it particularly effective in systems with higher proportions of apolar phase. PEG 100 Stearate represents a different structural approach with its polyethylene glycol polar group. Its high HLB value and strong affinity for the polar phase stem from the extensive ethoxylation of its structure. This unique molecular architecture enables the formation of versatile mixed systems (oil-in-water or water-in-oil), with the PEG chains providing steric stabilization at the interface. The surfactant’s ability to prevent solvent migration and facilitate the controlled release of active components can be attributed to the formation of a dense interfacial layer created by the PEG chains.

The optimal surfactant concentration of 10% identified in the study represents the critical concentration necessary for effective interfacial stabilization. At this concentration, the surfactant molecules can form a complete protective layer at the polar–apolar interface, preventing coalescence and maintaining system stability.

The correlation between HLB values and system stability demonstrates the importance of matching surfactant characteristics to specific application requirements. Low HLB surfactants (Span 60) excel in forming highly viscous gels with strong interfacial stability, moderate HLB surfactants (Cetyl Alcohol) provide balanced emulsion stability with medium viscosity, and high HLB surfactants (PEG 100 Stearate) offer versatility in mixed systems with controlled release properties. This structure–property relationship analysis confirms that surfactant selection must be based on careful consideration of molecular architecture, including chain length and polar group characteristics, in relation to the specific requirements of the target system. The results demonstrate that while there is no universal surfactant solution, understanding these molecular-level interactions enables rational selection for specific applications.

## 3. Conclusions

This study demonstrates that gels based on non-polar solvents and cellulose derivatives represent a valid ecological alternative for cleaning metallic artworks, opening new perspectives for more sustainable restoration practices that respect the health of operators and the environment.

Overall, the experimental study and subsequent formulation of the gels demonstrated that the careful selection of surfactants, based on parameters such as HLB and chemical compatibility (using HSP and RED calculations), plays a crucial role in the stability and rheological properties of oil-in-water emulsions. The stability of the formulated systems was confirmed by turbidity, rheological, and thermogravimetric analyses. The different gel formulations achieved excellent results in removing the various types of protective coatings applied to the laboratory-prepared specimen. The goal of the experiment has thus been achieved, namely, the formulation of stable gels containing an apolar phase within a gel matrix made from cellulose ethers, thereby limiting the interaction between the solvent and the operator, as well as between the solvent and the substrate. Future research aims to evaluate the interactions of the systems that performed best in this study with other types of natural thickeners, such as Gellan and Nevek. Furthermore, an additional step toward an increasingly green and sustainable process will involve evaluating the possibility of employing bio-surfactants within the formulations.

## 4. Materials and Methods

### 4.1. Gel Formulation and Property Definition

In the preliminary phase of the study, toxic organic solvents commonly used in restoration treatments were identified through an online survey designed and administered to professionals within the YOCOCU APS network. A total of 32 professionals, representing various ages and backgrounds, participated in the survey.

The selection of surfactants was based on the HLB system, considering that the emulsions produced are not influenced by formulation parameters, which were kept constant, such as surfactant concentration, temperature, and salinity. Different HLB values (4, 8, 12, 16) were evaluated to obtain stable oil-in-water (O/W) and water-in-oil (W/O) emulsions, drawing on data from the literature.

Ligroin was chosen as the non-polar component due to its being the least polar solvent among those used in the experiments and was purchased from CTS Conservation, Rome, Italy. As the polar component, a thickening agent and a co-surfactant were considered. The selection of these substances, as well as the surfactant, was based on the RED calculated using HSP through the HSPiP software (version 6). The RED value allows for the evaluation of the chemical affinity between two molecules and predicts the miscibility between two compounds [32].

The results of the RED calculations identified Klucel-G as the continuous phase in the experiment, while 1,2-hexandiol was selected as the co-surfactant. Klucel-G is the trade name of hydroxy propyl cellulose, which was purchased from IMAR Italy. All other products used in the experiment are of pure grade (>98%) and supplied by Thermo Fisher Scientific.

The co-surfactant was employed to reduce interfacial tension, enhance the clarity of the solution, and act as a regulator of both the surfactant’s polarity and hydration. This effect also minimized the amount of surfactant required.

Additional criteria for surfactant selection included volatility and boiling point. Another critical factor was toxicological safety, as residual solvent traces in the final product limit the range of suitable solvents.

To determine the most stable emulsions, different scans were performed by varying the proportions of the non-polar and polar phases as follows: 100%–0%, 75%–25%, 50%–50%, and 25%–75%, observing the macroscopic formation of deposits. The surfactant concentration was kept constant for all scans, while the cellulose ether was used in a limited amount (0.5% *w*/*w*) to obtain low-viscosity gels.

All the scans were analyzed using a Lovibond TB350 turbidimeter (Lovibond^®^ Water Testing, Schleefstraße 8-12,44287, Dortmund, Germany) to identify the mixtures with the highest transparency. Measuring turbidity was crucial for evaluating the stability and uniformity of the emulsions or the gels, as a low turbidity level indicates a more homogeneous dispersion and a clearer system, which is desirable for cleaning applications.

The stability of the emulsions was characterized using the Static Multiple Light Scattering (SMLS) technique (Turbiscan DnS, Formulaction). The stability of the different gels used in the experimentation was evaluated, using a gel of Klucel-G and an aqueous solution, which was considered stable by the restorers, as the reference [12].

The Turbiscan plots highlight the variation in transmission along the height of the sample during the measurement time. In a stable dispersion, the transmission profile does not undergo significant changes over time; conversely, an unstable dispersion will exhibit variations that indicate phenomena such as aggregation, sedimentation, or creaming. A total of 11 samples were analyzed: 10 gel samples formulated during the experiment (Gel 1–10) and the reference gel.

Additionally, viscosity measurements were conducted to assess the flow properties of the gels. Viscosity is a crucial parameter that influences the application and performance of gels in cleaning treatments, as it affects how easily the gel can be applied to surfaces and its ability to adhere to various materials.

By analyzing the viscosity of the formulated gels, we can optimize their consistency for practical use, ensuring they provide adequate coverage and stability during application. Viscosity measurements were conducted using a Digital Viscometer LB 22DV (Labtronics Scientific, Wilmington, NC, USA), operating in its standard configuration. The device has a measurement range of 20 to 2,000,000 mPa·s and offers a variety of rotational speeds (RPMs): 0.3, 0.6, 1.5, 3, 6, 12, 30, and 60. The viscometer uses four rotors—#1, #2, #3, and #4—to measure viscosity across different formulations. The instrument’s accuracy is ±1.0% full scale, with a repeatability of ±0.5% full scale.

Finally, thermogravimetric analysis (TGA) was employed to study the thermal stability of the gel formulations, with the primary aim of evaluating the solvent release capabilities by monitoring solvent loss as a function of temperature. This method provided insights into the evaporation behavior of the solvents, the decomposition of components, and any thermal transitions within the gel. These observations are crucial for understanding how the gels will perform during the cleaning process, particularly in terms of controlled solvent release. The TGA measurements were performed with a Mettler Toledo TGA2 instrument (Columbus, OH, USA), in an inert atmosphere, with nitrogen (N_2_) flowing at a rate of 60 mL/min. The temperature range for the analysis was 30–500 °C, with a heating rate of 10 °C/min, ensuring a gradual thermal increase to observe all relevant transitions. The samples were placed in an alumina crucible (70 μL capacity), ensuring precision in monitoring weight loss throughout the experiment.

### 4.2. Cleaning Tests

The gels that showed the best results in the previous phases of the experiment were applied to a copper frame (size 47 × 58 cm) coated with protective layers commonly used for the preservation of metal artifacts. The apolar protective coatings, to apply on a frame, were chosen based on the literature [33,34,35,36,37] to simulate two typical scenarios: one for the protection of indoor bronze artifacts, and the other for bronze statues exposed to an outdoor environment. Additionally, a distinction was made between single-layer and multi-layer protective coatings. The double-layer system involves the application of an intermediate layer between the metal surface and the protective coating. This intermediate layer, typically made of resin (acrylic, ketonic, or nitrocellulose), is usually loaded with corrosion inhibitors and serves to protect the metal without directly applying the final wax layer to its surface. Table 10 reports the products used in the study to realize the protective layers.

**Figure 12:**
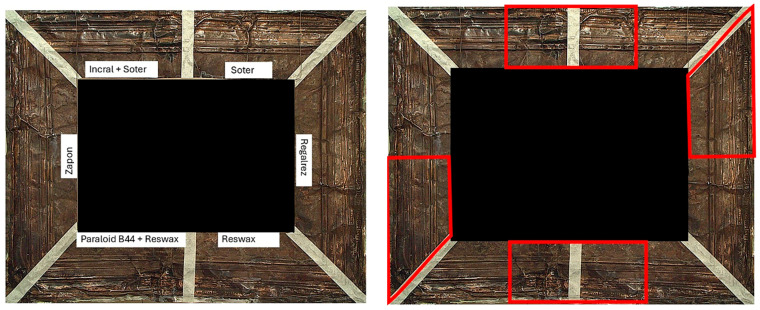
Oxidated copper frame with the application of products as described in Table 10. Sx: all surfaces; dx: area subjected to aging, in particular the red frames indicates the area that were covered during the aging period.

The copper frame was first cleaned and then treated with a 5% NaOH solution to form a patina of tenorite (CuO), a black copper oxide often used to simulate aged metal surfaces [36]. The prepared surface was then treated with various protective layers as outlined in Table 10. These products were applied by brush to specific areas, as shown in Figure 12. For each treatment, half of the area was sealed from the environment using a polyethylene film (Figure 12), while the entire frame was placed in a climatic chamber for accelerated aging in accordance with the Italian Standard UNI EN 16474—2014 [38]. This artificial aging process was applied to mimic realistic environmental conditions, allowing the efficacy of the various gels to be tested on both aged and unaged protective coatings.

The application of the protective layers followed typical methods for bronze conservation, with particular attention to the difference in performance before and after aging.

The formulated gels that showed the most promising results in previous phases were employed to remove the protective layers applied to the copper frame. The gels are synthesis in Table 11.

As a reference for the cleaning tests, Wax Rescue, a product from Lab4Green (www.lab4green.it, accessed on 14 June 2024), was used. According to the technical data sheet, this product is specifically designed for the removal of apolar substances in cultural heritage treatments and can be gelled using Klucel-G. This solvent was selected to benchmark the performance of the formulated gels against a commercially available solution commonly employed for similar cleaning purposes. The gels were left in contact with the surfaces for 5 min.

To evaluate the cleaning efficacy of the different tested gels, the following analyses were used:

(a) Multispectral imaging before and after the application of the various protective layers, following sample aging in the climatic chamber, and after the cleaning tests. The images were captured using a full-spectrum Canon EOS M50 camera (Canon, Tokyo, Japan), equipped with specific filters: the HOYA UV-IR filter (Madatec, Milan, Italy) with 52-cut, and the Yellow 495 52 mm F-PRO MRC 022 filter (Madatec, Milan, Italy). UV light source employed a wavelength of 365 nm.
(b) Digital optical microscopy observations using a DinoLite AM411-FVW Digital Microscope (Leica Microsystems, Wetzlar, Germany), operating at 40× and 220× magnification in the visible (VIS) and ultraviolet (UV) ranges. This allowed for a detailed study of the surface of the frame before and after the cleaning tests. Additionally, this analysis enabled us to observe potential residues of the protective coating or gel on the surface.
(c) To evaluate the color changes induced by the application and the removal of the protective layers. In this context, the ΔE value, a parameter that indicates the color difference, was calculated. ΔE values below 5 are considered imperceptible to the human eye [37]. Spectrocolorimetry was used to assess the color variations induced by the removal of the protective layer. In this context, the ΔE parameter was calculated between the original surface, prior to the application of the protective coatings, and the surface after the cleaning treatments. Spectrocolorimetry was performed using a YS3010 3nh Handheld Spectrophotometer (Shenzhen ThreeNH Technology Co., Ltd., Shenzhen, China).
(d) Fourier Transform Infrared Spectroscopy (FTIR) was performed in ATR mode with the Nicolet Summit Pro (Thermo Scientific, Waltham, MA, USA). This analysis was conducted in areas where the microscope observation suggested the presence of gel residues. The FTIR analysis allowed us to verify the residues (gel, solvent or protective coating) left on the surface. Spectra were acquired in the range of 400–3200 cm^−1^, with a total of 32 scans at a resolution of 4 cm^−1^.

## Figures and Tables

**Table 1:** Calculation of the RED value between the different cellulose ethers based. The table shows the HSP and the RED value and the Standard Deviation (SD).

Cellulose Ethers	Formula	δD (SD < 0.4)	δP (SD < 0.2)	δH (SD < 0.5)	RED
Klucel-G (Hydroxypropyl Cellulose)	C_36_H_70_O_19_	17.6	10.2	15.4	0
Hydroxypropylmethyl Cellulose (HPMC)	C_56_H_108_O_30_	17.4	8.9	15.9	0.18
Methyl Cellulose	C_20_H_38_O_11_	17.6	8.5	17	0.29
Idroxy Propyl Cellulose	C_3_H_7_O	17.5	8.1	16.3	0.29
Ethylhydroxypropyl Cellulose (EHPC)	C_30_H_56_O_19_	17.1	8.1	14.1	0.33
Hydroxypropyl Cellulose (HPC)	C_36_H_70_O_19_	17.6	9.9	18.6	0.4
Cellulose Acetate (Celidora A, R Cellulose Acetate)	[C_6_H_7_O_2_(OCOCH_3_)_3_]_n_	18.2	12.4	10.8	0.65
Ethyl Cellulose (Ethocel Std 20, Ethocel He10)	C_12_H_22_O_5_(C_2_H_5_)_n_	16.8	7.2	11.5	0.65
Carboxymethyl Cellulose	C_6_H_7_O_2_(OH)_x_(OCH_2_COOH_)y_	18.1	10.7	21.3	0.75
Nitrocellulose	[C_6_H_7_O_2_(ONO_2_)_3_]_n_	15.41	14.73	8.84	1.14
Cellulose Acetobutyrate	C_6_H_7_O_2_(OCOCH_3_)_x_(OCOC_3_H_7_)_y_	16.6	12	6.7	1.14
Carboxymethylhydroxypropyl Cellulose (CMHPC)	C_6_H_7_O_2_(OCH_2_COOH)(OC_3_H_6_OH)_n_	18	11.6	29.5	1.77
Hydrophobically Modified Hydroxyethyl Cellulose (HMHEC)	Complex formula, varies based on modifications	25.9	6.4	14.4	2.13
Hydroxyethylpropyl Cellulose (HEPC)	C_6_H_7_O_2_(OH)(OC_2_H_4_OH)_x_(OC_3_H_6_OH)_y_	26.3	7.4	13.9	2.21
Methylhydroxyethylhydroxypropyl Cellulose (MHEHPC)	Complex, varies (methyl + hydroxyethyl + hydroxypropyl)	28.5	9.5	18.5	2.75
Ethylhydroxyethyl Cellulose (EHEC)	C_6_H_7_O_2_(OH)(OC_2_H_4_OH)_x_(OC_2_H_5_)_y_	29	8.3	15.5	2.86
Carboxymethylhydroxyethyl Cellulose (CMHEC)	C_6_H_7_O_2_(OH)_x_(OCH_2_COOH)(OC_2_H_4_OH)	28.2	10.9	31	3.29

**Table 2:** RED between the possible different surfactants chosen based on HLB values versus the apolar phase of the system (Ligroin). SD < 0.2.

Surfactant	δD	δP	δH	RED	HLB
Ligroin (Reference)	16	0	0	0	-
Cetyl Alcohol	16.1	3	6.1	0.85	15.5
Cetearyl Alcohol	16.1	3	6.35	0.88	15.5
PEG 100 Stearate	16.02	2.53	6.6	0.88	18
Stearyl Alcohol	16.1	3.1	6.6	0.91	15.5
Tefose 63	16.3	3.4	6.6	0.93	9.7
Dodecylphenol Ethoxylate (6)	16.6	5	7.4	1.13	10.00
Polyoxyethylene (20) Sorbitan Tristearate	16.8	5.2	7.3	1.14	11
Polyoxyethylene (20) Sorbitan Trioleate Tween 85	16.6	5.3	7.5	1.16	11
Dodecylphenol Ethoxylate (12)	16.8	5.8	7.5	1.2	13–14
Nonylphenol Ethoxylate (30)	16.8	6.7	7.3	1.25	15–16
Isostearyl Monoethanolamide	16.9	6.4	8.5	1.35	4–6
Polyoxyethylene (20) Sorbitan Monostearate	16.8	6.4	9.4	1.44	15
Polyoxyethylene (20) Sorbitan Monooleate Tween 80	16.7	6.5	9.4	1.44	14.9
Polyoxyethylene (20) Sorbitan Monopalmitate	16.8	6.5	9.5	1.45	15.6
Polyoxyethylene Sorbitan Monolaurate (Tween 20)	16.7	6.7	9.7	1.48	16.7
Stearyl Monoethanolamide	15.8	5.6	10.4	1.48	5–8
Oleyl Diethanolamide	15.8	5.6	10.4	1.48	5–6
Sorbitan Monoleate (Span 80)	16.7	6.7	9.7	1.48	4.3
Polyoxyethylene (5) Sorbitan Monooleate Tween 81	16.6	6.1	10.5	1.53	10
Polyoxyethylene (4) Sorbitan Monostearate	16.8	6.1	11	1.58	9.6
Lauryl Monoethanolamide	16.8	7.6	10	1.58	5–7
Lauryl Diethanolamide	16.9	6.4	11.9	1.7	8–10
Polyoxyethylene (4) Sorbitan Monolaurate	16.5	6.6	12	1.72	13.3
Capric Diethanolamide	16.3	6.6	12.2	1.74	8–10
Sorbitan Monostearate (Span 60)	17.1	6.2	12.4	1.75	4.7
Sorbitan Monopalmitate (Span 40)	17.1	6.3	12.7	1.79	6.7
Diethylene Glycol Monoethyl Ether	16.1	9.2	12.2	1.91	-
Sorbitan Monolaurate (Span 20)	16.7	6.8	13.7	1.92	8.6

**Table 3:** RED between the different surfactants versus the polar phase of the system (Klucel-G + 1,2-hexanediol). SD < 0.2.

Surfactant	δD	δP	δH	RED
1,2-Hexanediol + Klucel-G (83:17)	16.3	7	13.3	0
Sorbitan Monolaurate (Span 20)	16.7	6.8	13.7	0.11
Sorbitan Monooleate Span 80	16.7	6.1	12.4	0.19
Sorbitan Monopalmitate (Span 40)	17.1	6.3	12.7	0.23
Sorbitan Monostearate (Span 60)	17.1	6.1	12.3	0.26
Diethylene Glycol Monoethyl Ether	16.1	9.2	12.2	0.31
Methocel 311	17.3	9.9	13.5	0.44
Polyoxyethylene (20) Sorbitan Monolaurate (Tween 20)	16.7	6.7	9.7	0.46
Klucel H	17.6	10.2	15.4	0.58
Tefose 63	16.3	3.4	6.6	0.95
Cetyl Alcohol	16.1	3.1	6.6	0.97
PEG 100 Stearate	16.1	3	6.35	0.98
Stearyl Alcohol	16.1	3	6.1	1.03
Wax Rescue	14.7	3.43	5.9	1.1
Methyl Caprate	15.9	2.4	5.7	1.11
Methyl Oleate	16.2	3.8	4.5	1.17
Methyl Laurate	16	2.1	5.2	1.19
Isopropyl Palmitate	16.2	3.9	3.7	1.26
Methyl Cocinate	16	1.8	4.7	1.26
Methyl Canolate	14.8	2.7	4.5	1.28
Methyl Miristate	16	1.9	4.2	1.31
Methyl Soiate	16.1	1.6	3.8	1.37
Methyl Palmitate	16	1.6	3.6	1.39
Methyl Stearate	15.9	1.4	3.2	1.45
HPMC	19.3	14.1	20.6	1.5
Ligroina	16	0	0	1.88
Water	15.5	16	42.3	3.8

**Table 4:** Determinantion of surfactants concentration for the formulation of a stable O/W emulsion. Turbidity measurements expressed in NTU.

0.5 g		1 g		1.5 g		2 g		2.5 g	
Average	SD	Average	SD	Average	SD	Average	SD	Average	SD
4.27	2.04	3.09	0.95	3.43	1.40	8.11	0.27	9.93	0.93

**Table 5:** Turbidity measurements, expressed in NTU, acquired for the various Ligroin/(Klucel-G + 1,2-Hexanediol) emulsions with the addition of different surfactants.

% Apolar/Polar Phases	PEG 100 Stearate		Span 60		Cetyl Alcohol		Tefose^®^ 63		Mix Tween 20/Span 80	
	Average	SD	Average	SD	Average	SD	Average	SD	Average	SD
100	108	2.9	3.1	0.1	110	2.1	160	2.3	deposit	-
75/25	59	0.7	2.26	0.5	97	2.8	86	1.8	deposit	-
50/50	21	0.3	27.7	1.5	21	1.9	40	1.6	62	1.3
25/75	12	1.4	12.2	1.6	19	1.1	13	0.5	35.8	1.8
100	4.5	0.3	5.1	0.5	11	0.9	10	0.3	39.6	3.1

**Table 6:** Gels used in the laboratory cleaning of prepared samples.

ID Number	Apolar Phase	Surfactant
1	Ligroin	PEG 100 Stearate
2	Ligroin	Cetyl Alcohol
3	Ligroin	Span 60
4	Methyl Myristate	PEG 100 Stearate
5	Methyl Myristate	Cetyl Alcohol
6	Methyl Myristate	Span 60
7	Isopropyl Palmitate	PEG 100 Stearate
8	Isopropyl Palmitate	Cetyl Alcohol
9	Isopropyl Palmitate	Span 60

**Table 7:** Viscosity measurements of the different gels formulated. The Standard Deviation is reported as *a* percentage.

ID	Media (mPA·s^−1^)	SD (%)
Gel 1	290,321	1.8
Gel 2	181,266	2.8
Gel 3	106,704	3.9
Gel 4	284,421	1.7
Gel 5	134,381	1.1
Gel 6	213,629	2.7
Gel 7	264,725	1.8
Gel 8	272,425	0.6
Gel 9	201,655	3.5

**Table 8:** Images acquired of the copper frame at different electromagnetic spectrum regions.

	Vis	UV	UV Yellow CUT
Frame before the application of protective coatings	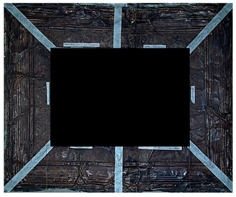	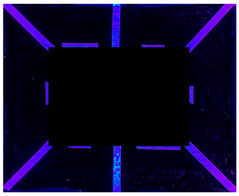	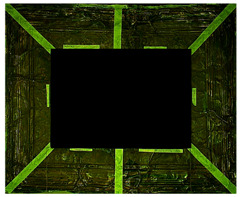
Frame after the application of protective coatings	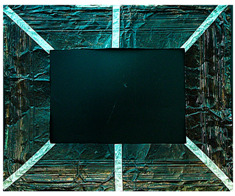	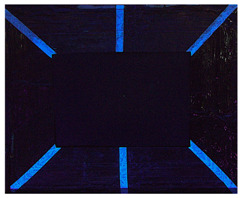	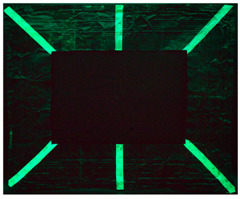
Frame after aging process	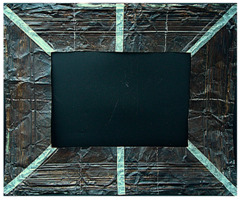	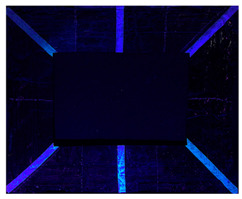	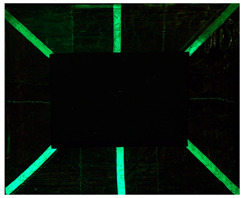
Frame after the cleaning tests.	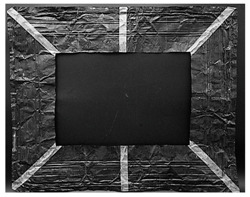	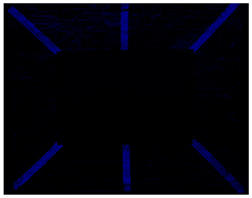	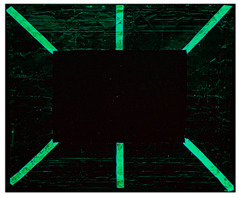

**Table 9:** ΔE between the chromatic parameter of the frame before the application of protective coatings vs. the frame after the cleaning treatment.

Coating		Gel 10	SD	Gel 8	SD	Gel 9	SD	Gel 1	SD	Gel 4	SD	Gel 7	SD	Gel 3	SD	Gel 6	SD	Gel 2	SD	Gel 5	SD
Regalrez	Not aged	2.7	0.25	4.5	0.43	7.2	0.28	4.5	0.22	3.6	0.32	3.0	1.26	2.6	0.1	2.5	2.43	3.9	0.31	2.5	1.25
	Aged	3.6	0.48	2.1	0.18	4.9	1.98	1.9	0.14	2.3	0.17	1.4	0.21	2.4	0.43	1.3	0.23	3.4	0.27	4.4	0.13
Reswax	Not aged	4.7	0.39	3.5	0.17	2.3	0.18	3.6	0.37	2.8	0.49	4.2	0.43	4.1	1.38	3.5	0.39	3.8	0.11	3.1	0.22
	Aged	3.5	0.34	2.0	1.62	3.1	0.31	3.5	0.28	5.1	2.41	4.0	0.24	4.8	0.39	2.3	1.36	3.7	0.14	2.9	0.16
Reswax + Paraloid B44	Not aged	3.5	0.16	4.5	0.22	4.2	1.7	2.0	0.15	3.5	0.48	3.8	2.21	4.1	3.41	2.8	0.45	6.7	0.11	2.3	2.47
	Aged	4.0	0.16	4.0	2.31	4.9	0.12	2.7	1.97	1.7	0.46	4.5	0.32	5.0	0.13	2.3	0.29	2.8	0.35	2.2	0.42
Zapon	Not aged	4.0	0.12	3.9	0.27	8.0	0.34	4.1	0.11	2.2	0.34	3.3	0.16	2.9	1.24	1.7	0.15	3.2	0.23	2.6	0.35
	Aged	4.2	0.45	3.7	0.22	5.8	0.17	4.1	0.46	5.9	0.47	4.0	1.42	2.7	0.15	3.0	0.39	3.0	0.3	1.7	2.45
Soter + Incral	Not aged	2.9	0.34	3.9	1.34	3.7	0.13	3.2	2.2	3.8	1.14	3.7	0.13	3.5	0.45	3.1	1.4	2.8	0.46	1.9	0.42
	Aged	4.8	0.38	4.9	0.16	4.1	0.48	2.6	0.37	2.9	1.18	5.0	0.49	3.1	0.35	2.7	0.32	3.8	0.2	3.3	0.17
Soter wax	Not aged	3.2	0.11	3.5	0.22	3.3	1.87	4.0	0.22	1.4	0.12	2.6	0.41	4.3	0.23	1.9	0.41	2.3	0.26	2.1	0.46
	Aged	2.4	0.49	2.2	0.25	3.4	0.42	4.2	0.31	4.0	0.23	3.3	0.18	3.2	0.13	4.4	0.3	2.0	0.4	3.8	0.32

**Table 10:** Products applied to simulate real-world applications on bronzes, both for indoor and outdoor. The commercial names are provided, as well as the use for applying either a single or double layer, along with the number of applications (layers).

Bronze	First Layer	No. of Applications	Second Layer	No. of Applications
Outdoor	Incral (acrylic varnish)	2	Soter (microcrystalline wax with inhibitors)	2
	Soter	2	Soter (applied to hot)	2
Indoor	Zapon (Nitrocellulose Lacquer)	2	no	-
	Regalrez (Hydrogenated Hydrocarbon Resin)	2	no	-
	Paraloid B44 (Acrylic Resin)	2	Reswax	2
	Reswax (Microcrystalline Wax)	2	no	

**Table 11:** Gels used for cleaning treatments divided for the apolar phase and the kind of surfactant used for the gel formulation. In all gels, the polar phase of the different gels consists of Klucel-G and 1,2-hexanediol.

ID Number	Apolar Phase	Surfactant
1	Ligroin	PEG 100 Stearate
2	Ligroin	Cetyl Alcohol
3	Ligroin	Span 60
4	Methyl Myristate	PEG 100 Stearate
5	Methyl Myristate	Cetyl Alcohol
6	Methyl Myristate	Span 60
7	Isopropyl Palmitate	PEG 100 Stearate
8	Isopropyl Palmitate	Cetyl Alcohol
9	Isopropyl Palmitate	Span 60

## Data Availability

The original contributions presented in the study are included in the article/Supplementary Materials, further inquiries can be directed to the corresponding author.

## Supplementary Materials

The following supporting information can be downloaded at: www.mdpi.com/article/10.3390/gels10110747/s1, Table S1. Observation under digital light microscopy on the frame area treated with Regalrez protector using Ligroin gels. Table S2. Observation under digital light microscopy on the frame area treated with Regalrez protective agent. Cleaning treatment using methyl myristate gels. Table S3. Observation under digital light microscopy on the frame area treated with Regalrez protective agent. Cleaning treatment using Isopropyl Palmitate gels. Table S4. Observation under digital light microscopy on the frame area treated with Reswax protective agent. Cleaning treatment using Ligroin, Methyl Myristate and Isopropyl Palmitate gels. Table S5. Observation under digital light microscopy on the frame area treated with the protective Reswax + Paraloid B44. Cleaning treatment using Ligroin, Methyl Myristate and Isopropyl Palmitate gels. Table S6. Observation under digital light microscopy on the frame area treated with Zapon protective. Cleaning treatment using Ligroin, Methyl Myristate and Isopropyl Palmitate gels. Table S7. Observation under digital light microscopy on the frame area treated with Incral + Soter multilayer. Cleaning treatment using Ligroin, Methyl Myristate and Isopropyl Palmitate gels. Table S8. Observation under digital light microscopy on the frame area treated with Soter’s protective agent. Cleaning treatment using Ligroin, Methyl Myristate and Isopropyl Palmitate gels.
