# Supplementary material for: Developing Innovative Apolar Gels Based on Cellulose Derivatives for Cleaning Metal Artworks
Source: Gels. 2024 Nov 16;10(11):747. doi: 10.3390/gels10110747 (PMC11593746; doi:10.3390/gels10110747)
Supplement: Supplementary file 1 [file gels-10-00747-s001.zip › gels-3296311-supplementary.pdf]

gels

Article

Developing innovative apolar gels based on cellulose derivatives for cleaning metalwork

Andrea Macchia[1], Camilla Zaratti[2,*], Davide Ciogli[3], Giovanni Rivici[4], Silvia Pilati[4], Nereo Sbiri[2], Tilde de Caro[5] and Maria Assunta Navarra[6]

[1] YOCOCU APS, Youth in Conservation of Cultural Heritage, Via Torquato Tasso 108, 00185 Rome; info@lab4green.it,
[2] YOCOCU APS, Youth in Conservation of Cultural Heritage, Via Torquato Tasso 108, 00185 Rome; aps@yococu.com
[3] YOCOCU APS, Youth in Conservation of Cultural Heritage, Via Torquato Tasso 108, 00185 Rome; davide.ciogli@emec.it
[4] YOCOCU APS, Youth in Conservation of Cultural Heritage, Via Torquato Tasso 108, 00185 Rome; laboratorio@beaumontitalia.it
[5] CNR-ISMN, Strada Provinciale 35 d n. 9, 00010 Rome; Italy
[6] Department of Chemistry, La Sapienza University of Rome, Piazzale Aldo Moro 5, 00185 Rome; mariassunta.navarra@uniroma1.it

Citation: To be added by editorial staff during production.

Academic Editor: Firstname Lastname

Received: date
Revised: date
Accepted: date
Published: date

Copyright: © 2024 by the authors. Submitted for possible open access publication under the terms and conditions of the Creative Commons Attribution (CC BY) license (https://creativecommons.org/licenses/by/4.0/).

gels

MDPI

Supplementary Materials

Table S1. Observation under digital light microscopy on the frame area treated with Regalrez protector using Ligorin gels.

REGALREZ	Aged		Not Aged	
Solvent: Ligroin				
Before cleaning	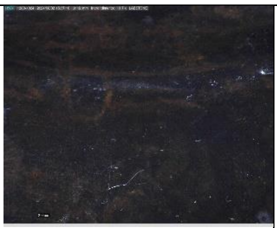	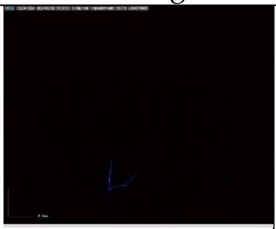	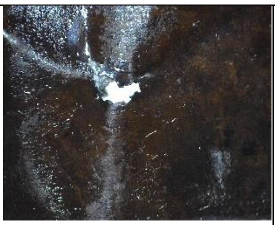	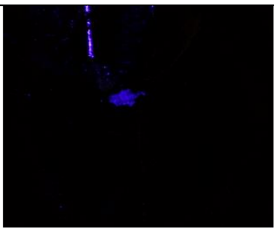
After cleaning	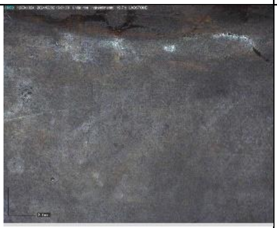	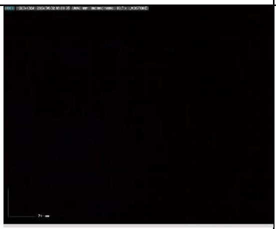	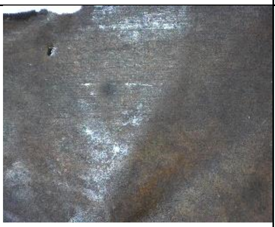	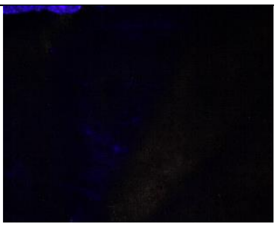
Before cleaning Gel 1	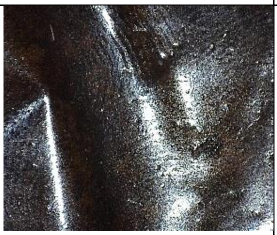	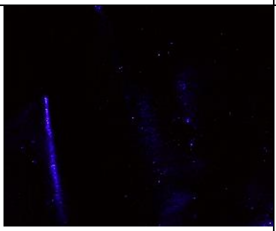	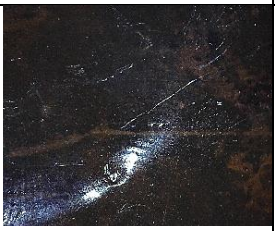	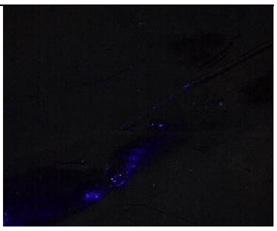
After cleaning	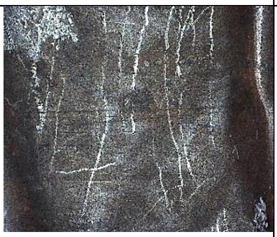	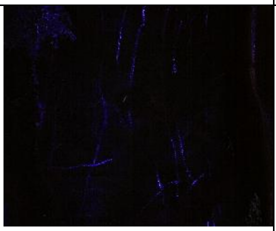	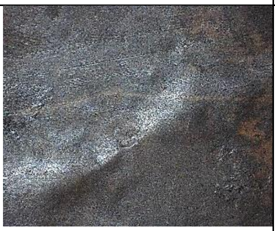	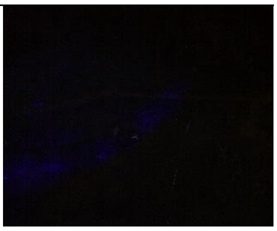
Before cleaning Gel 2	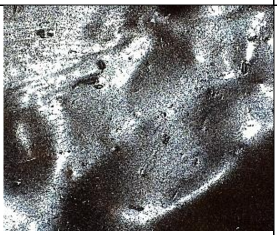	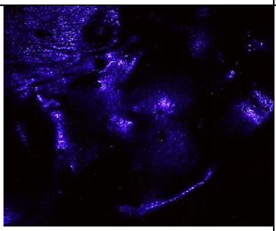	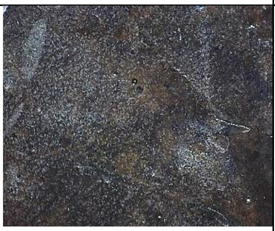	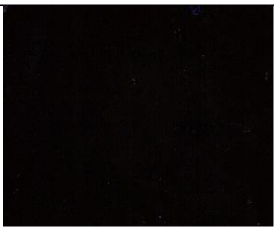

gels

MDPI

After cleaning	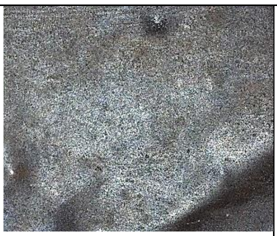	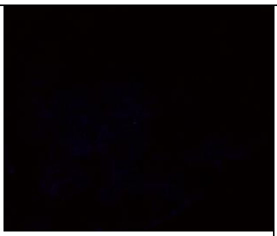	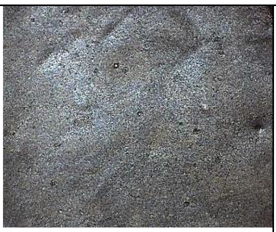	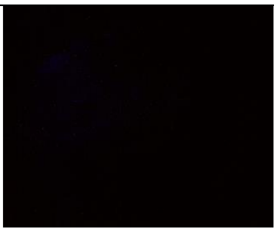
Before cleaning<br>Gel 3	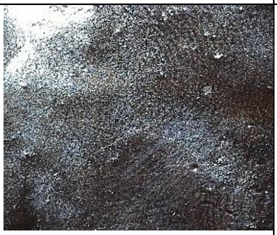	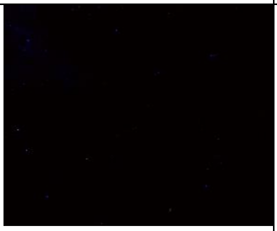	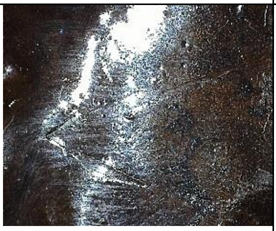	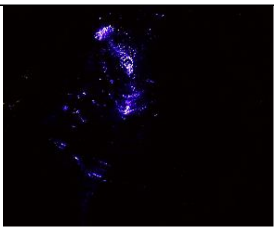
After cleaning	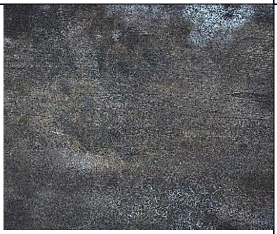	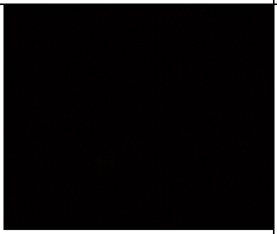	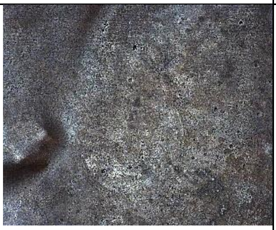	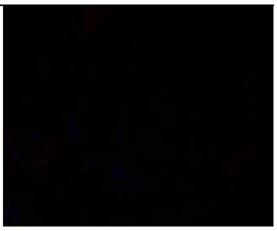

Table S2. Observation under digital light microscopy on the frame area treated with Regalrez protective agent. Cleaning treatment using methyl myristate gels.				
Methyl Myristate (MM)				
Before cleaning	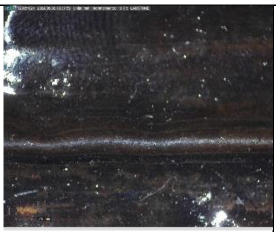	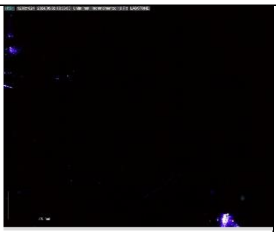	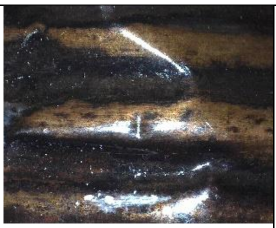	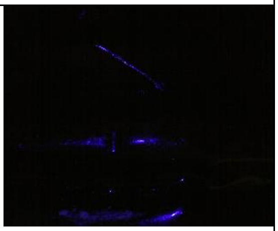
After cleaning	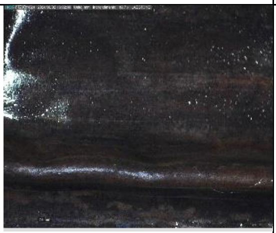	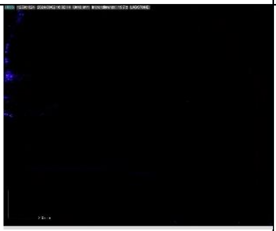	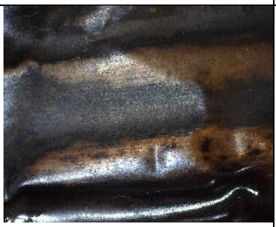	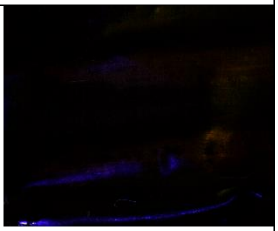
Before cleaning<br>Gel 4	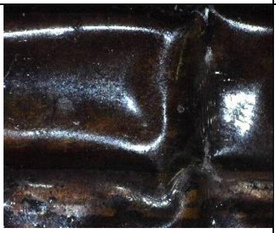	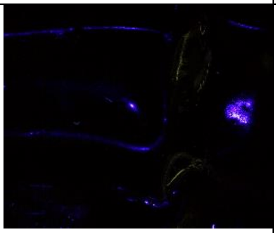	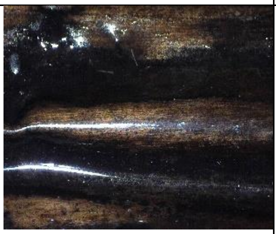	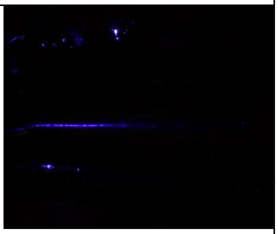

After cleaning	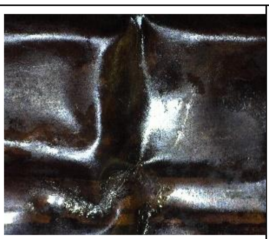	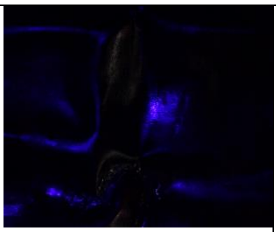	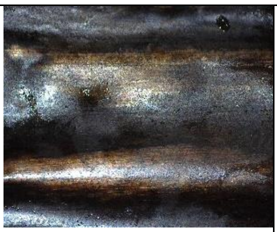	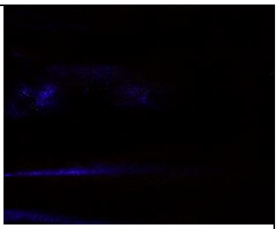
Before cleaning<br>Gel 5	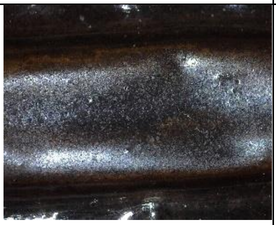	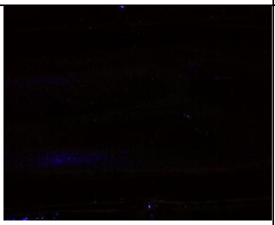	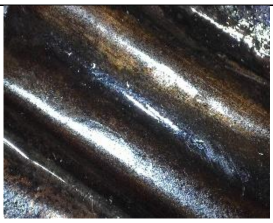	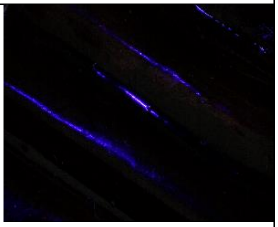
After cleaning	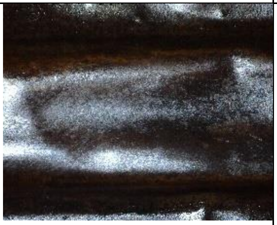	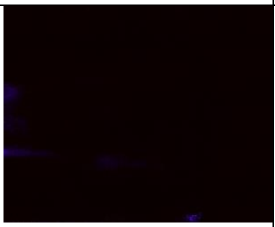	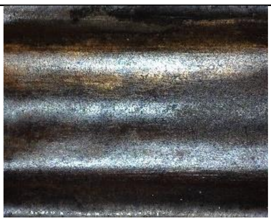	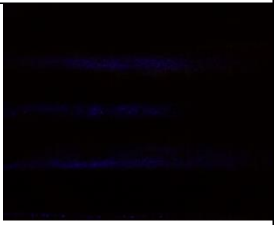
Before cleaning<br>Gel 6	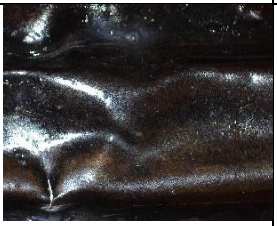	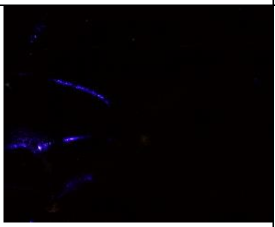	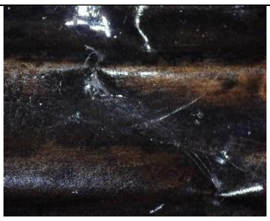	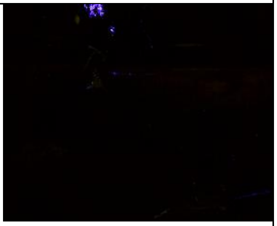
After cleaning	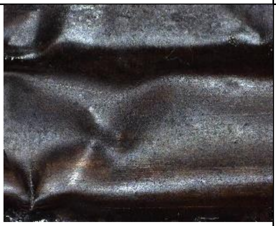	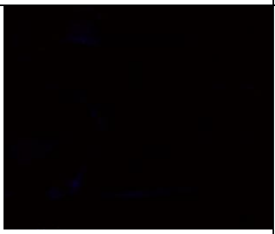	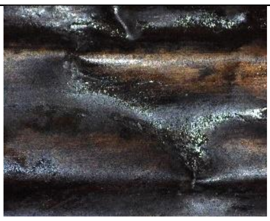	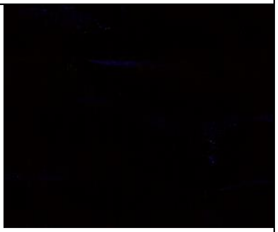

Table S3. Observation under digital light microscopy on the frame area treated with Regalrez protective agent. Cleaning treatment using Isopropyl Palmitate gels.				
ISOPROPYL PALMITATE (IPP)				
Before cleaning<br>IPP liquida	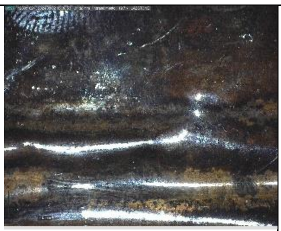	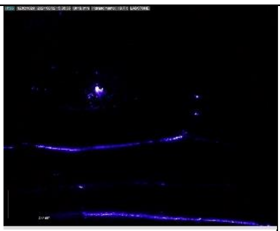	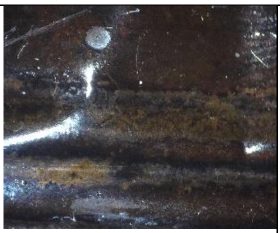	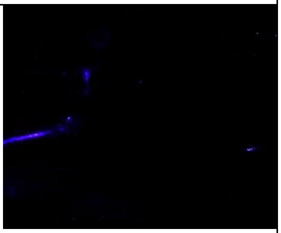

After cleaning	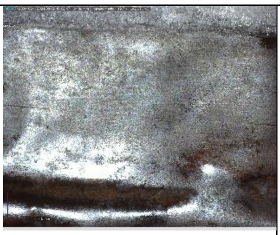	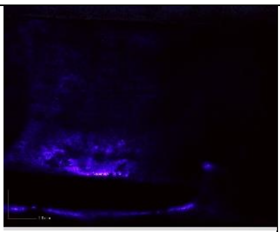	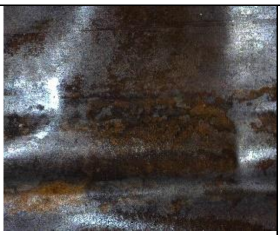	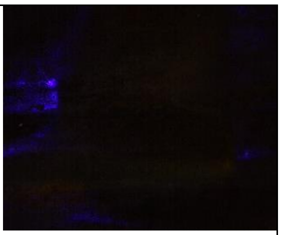
Before cleaning GEL 7	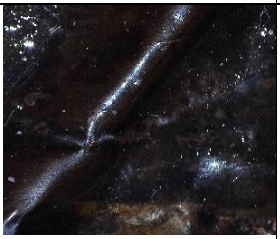	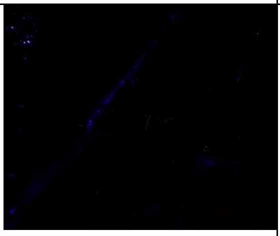	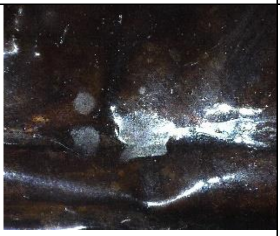	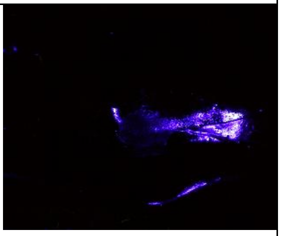
After cleaning	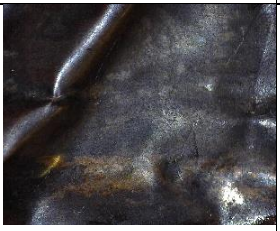	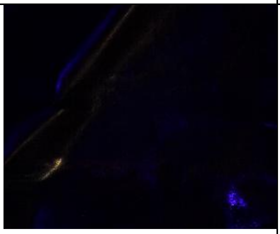	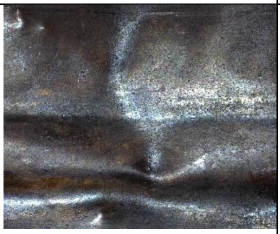	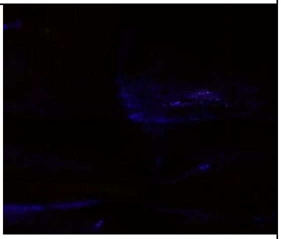
Before cleaning GEL 8	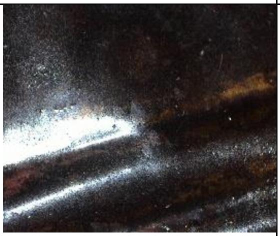	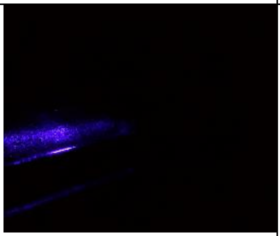	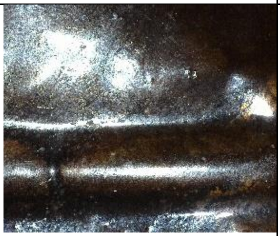	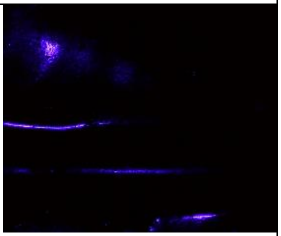
After cleaning	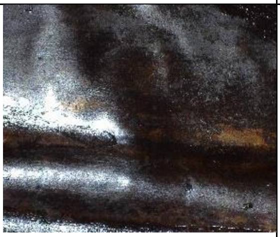	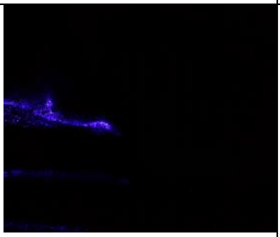	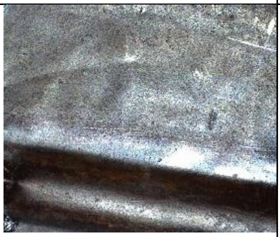	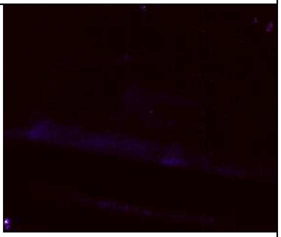
Before cleaning GEL 9	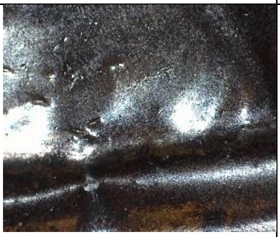	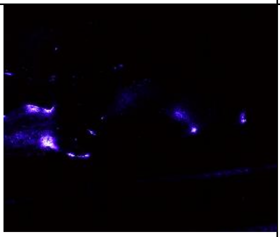	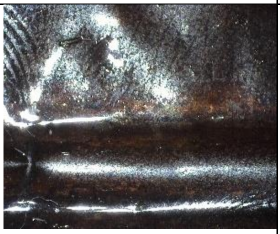	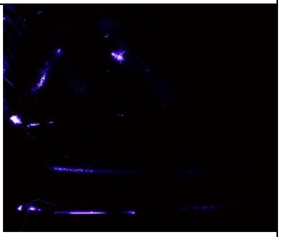
After cleaning	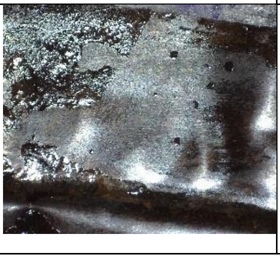	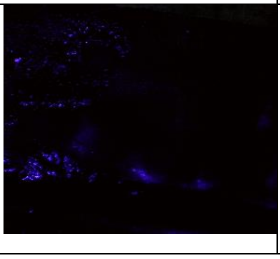	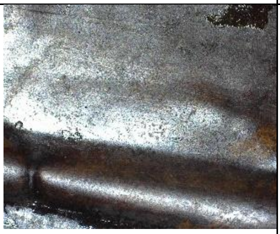	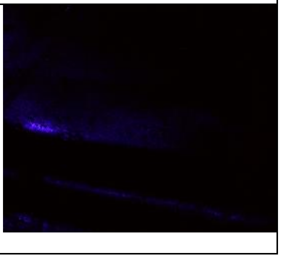

Table S4. Observation under digital light microscopy on the frame area treated with Reswax protective agent. Cleaning treatment using Ligroin, Methyl Myristate and Isopropyl Palmitate gels.

RESWAX	Aged		Not Aged	
Ligroin				
Before cleaning	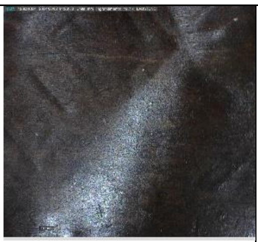	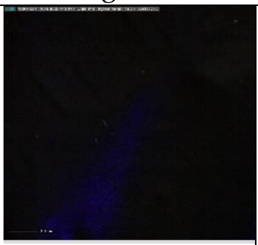	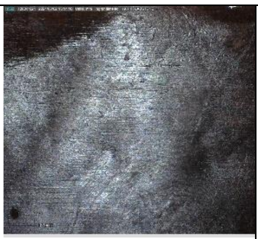	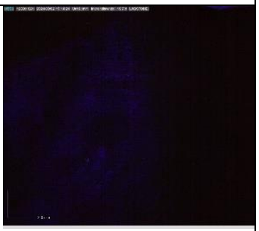
After cleaning	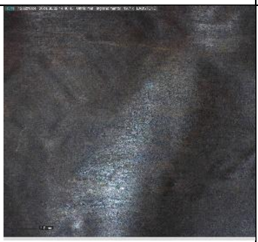	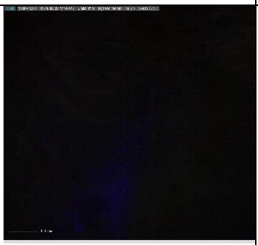	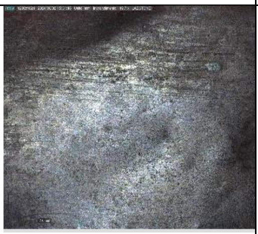	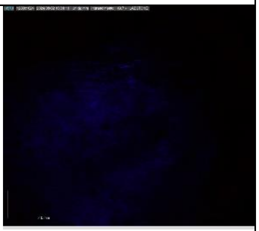
Before cleaning Gel 1	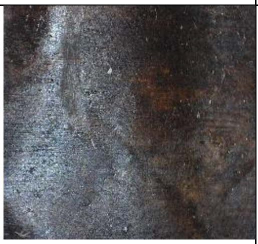	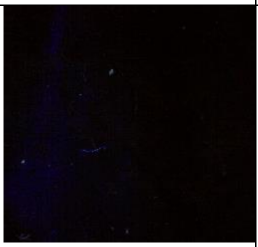	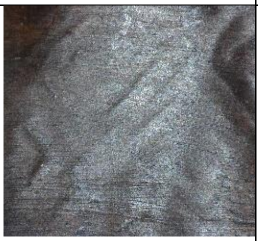	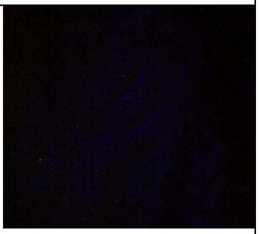
After cleaning	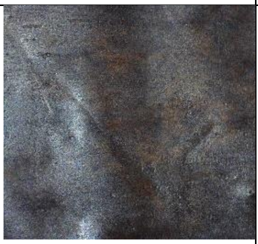	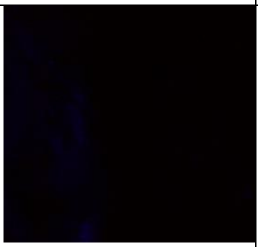	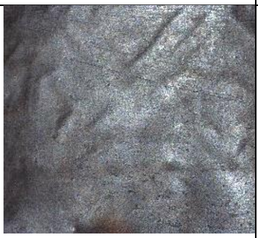	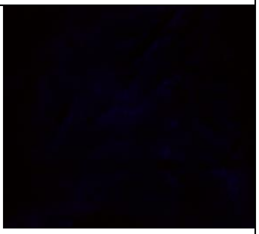
Before cleaning Gel 2	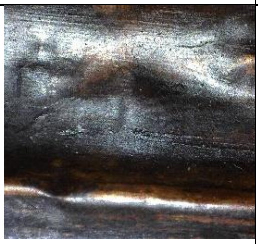	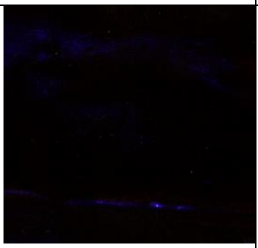	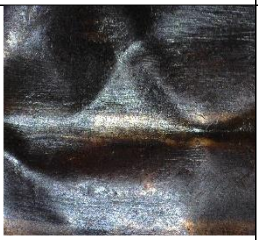	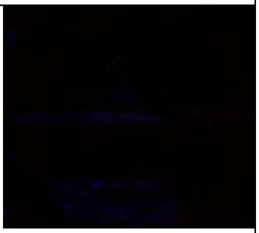
After cleaning	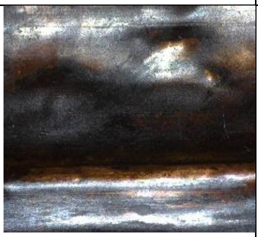	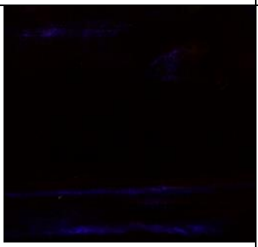	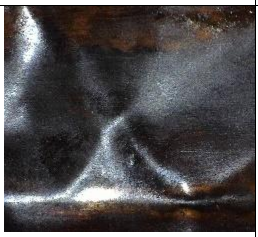	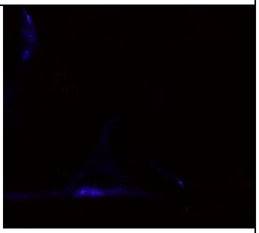

Before cleaning Gel 3	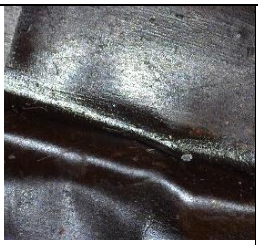	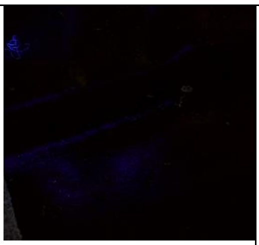	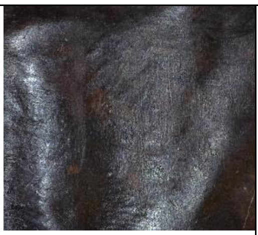	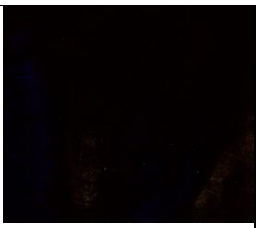
After cleaning	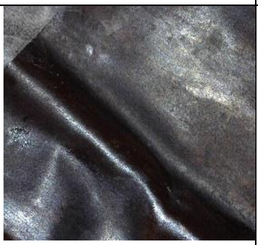	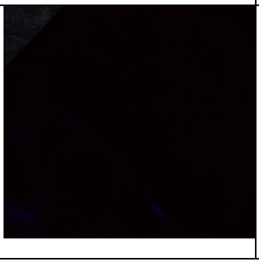	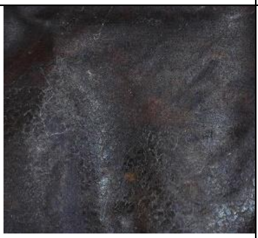	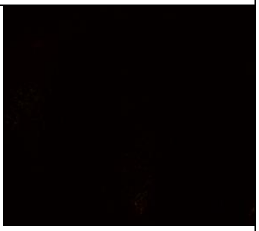
Methyl Myristate (MM)				
Before cleaning	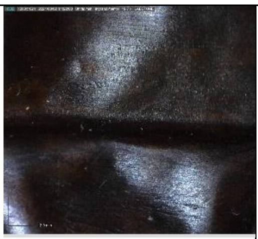	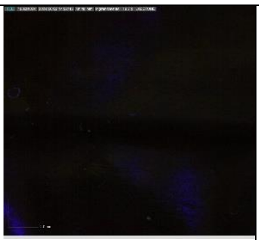	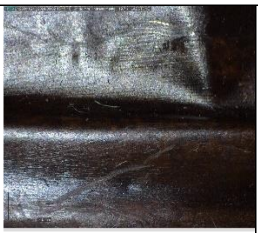	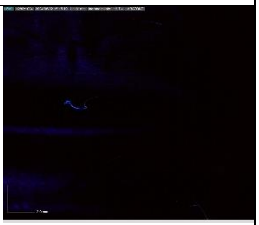
After cleaning	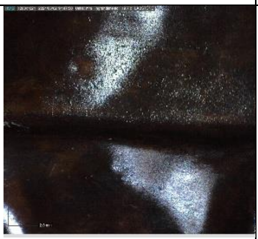	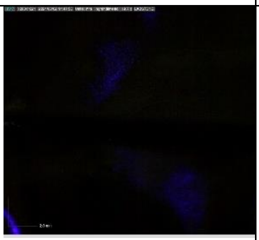	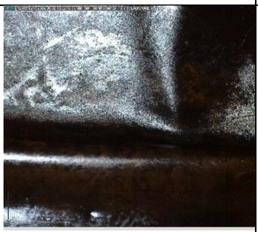	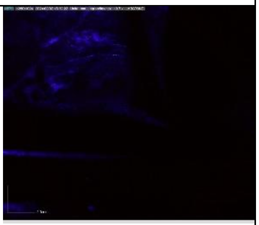
Before cleaning Gel 4	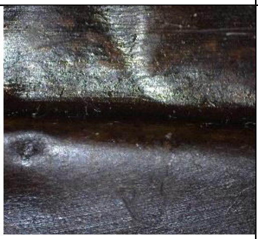	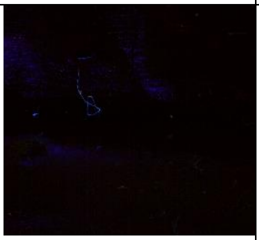	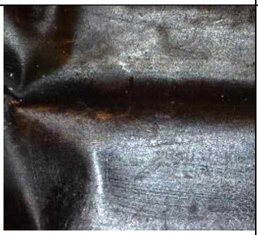	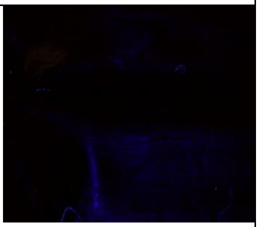
After cleaning	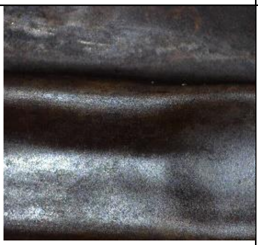	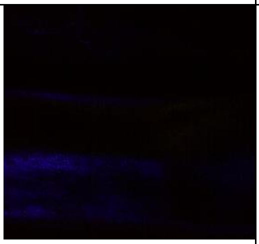	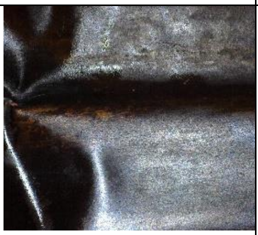	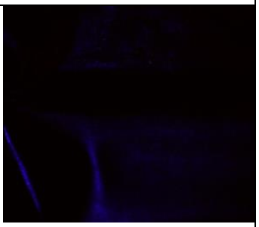

gels

Before cleaning Gel 5	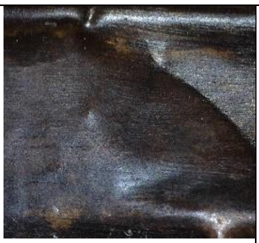	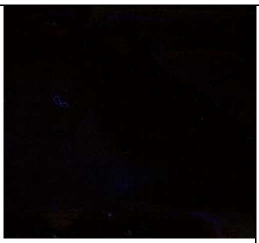	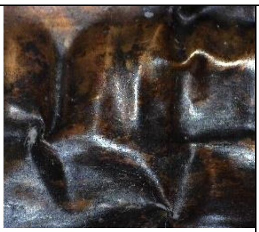	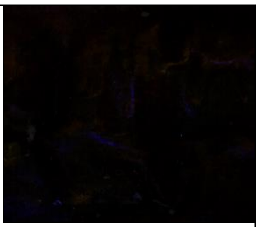
After cleaning	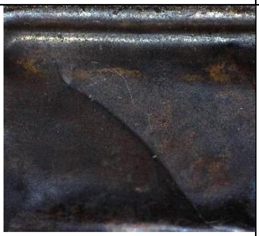	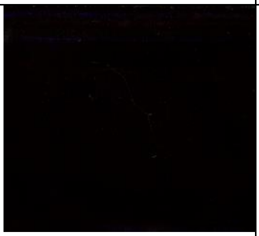	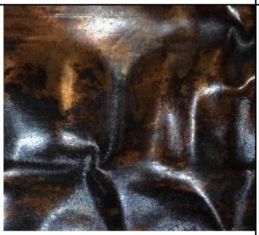	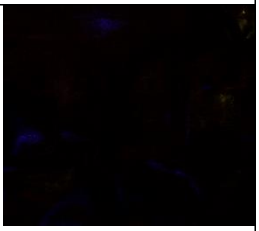
Before cleaning Gel 6	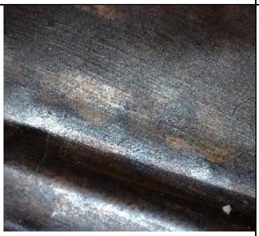	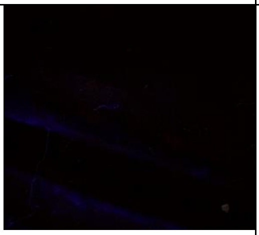	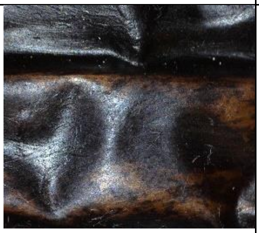	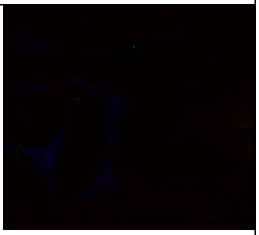
After cleaning	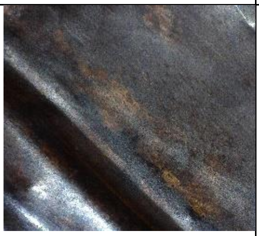	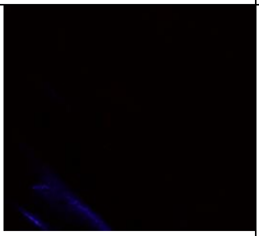	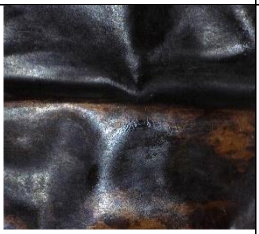	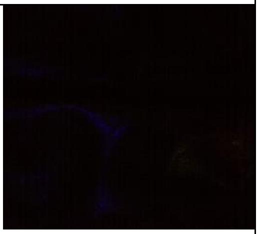
ISOPROPYL PALMITATE (IPP)				
Before cleaning	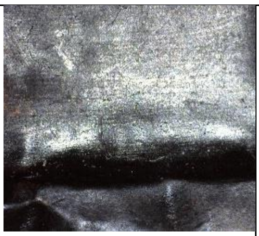	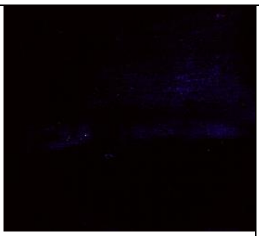	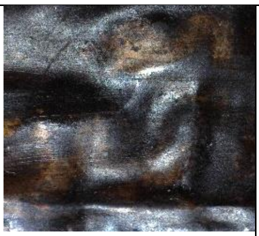	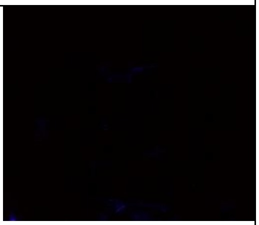
After cleaning	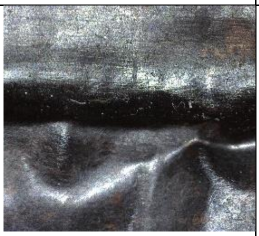	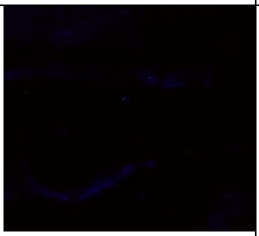	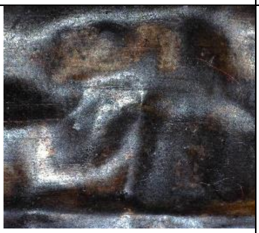	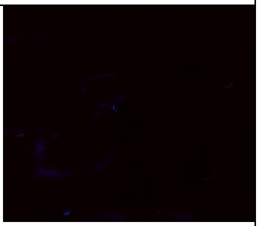

Before cleaning<br>Gel 7	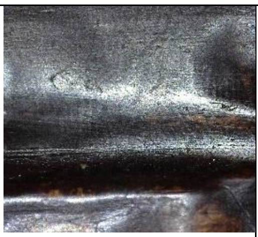	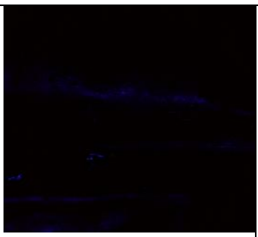	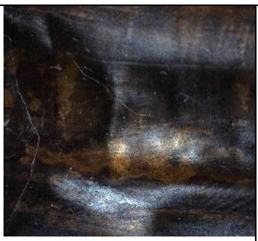	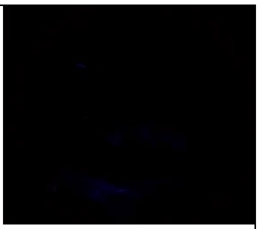
After cleaning	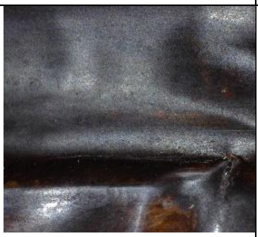	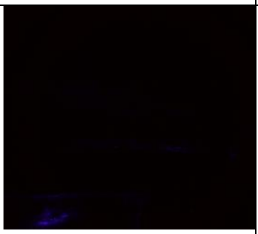	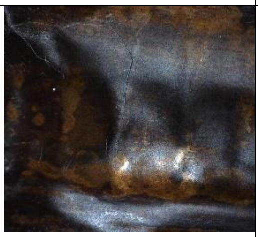	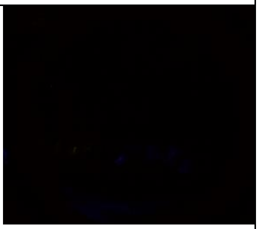
Before cleaning<br>Gel 8	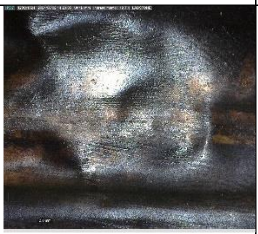	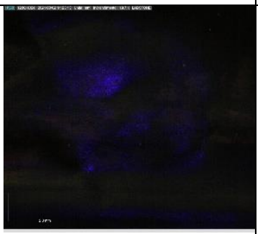	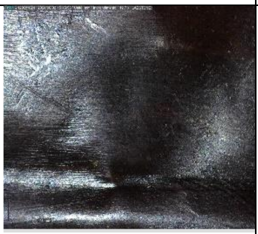	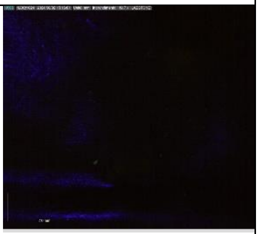
After cleaning	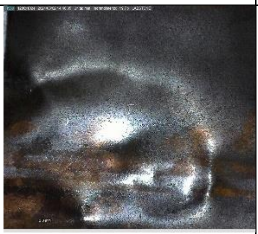	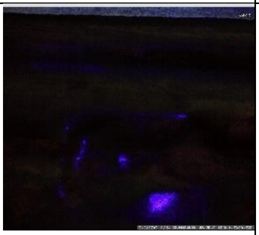	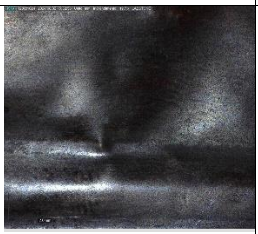	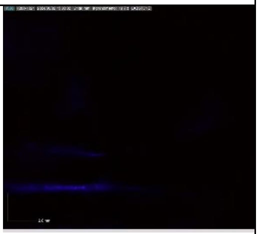
Before cleaning<br>Gel 9	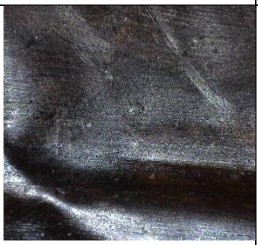	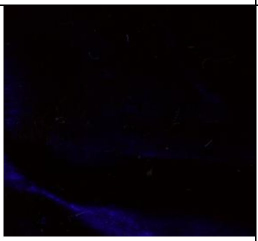	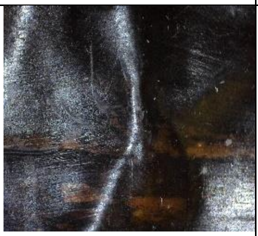	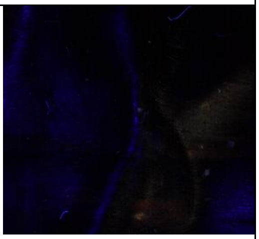
After cleaning	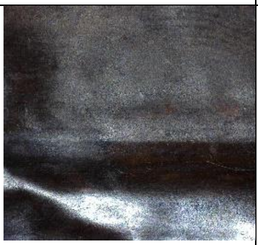	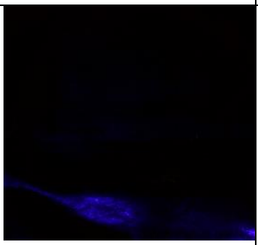	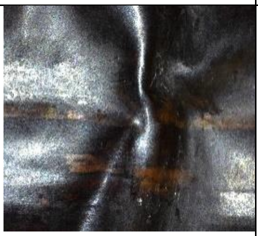	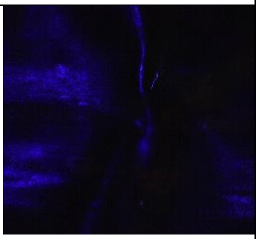

Table S5. Observation under digital light microscopy on the frame area treated with the protective Reswax + Paraloid B44. Cleaning treatment using Ligroin, Methyl Myristate and Isopropyl Palmitate gels..

RESWAX + PARALOID B44	Aged	Not Aged

gels

MDPI

Ligroin				
Before cleaning	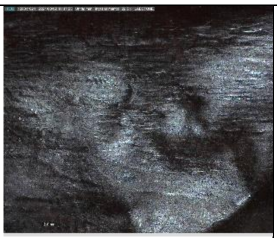	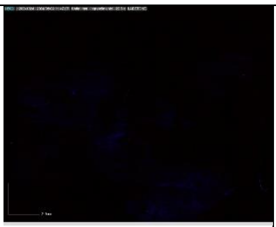	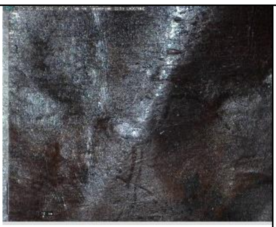	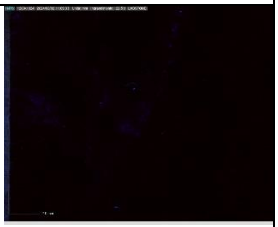
After cleaning	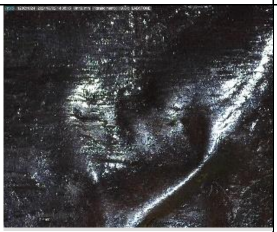	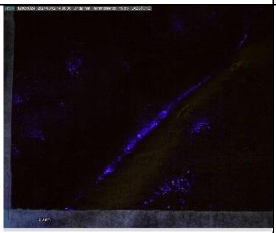	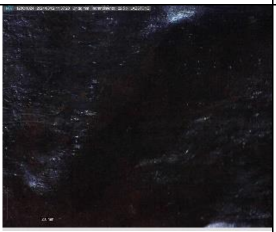	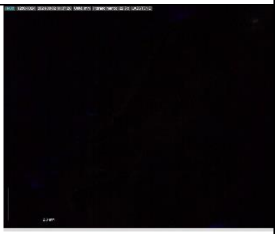
Before cleaning Gel 1	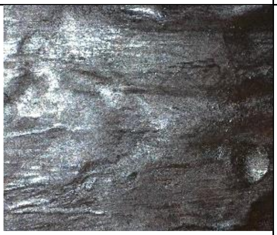	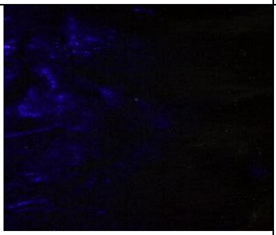	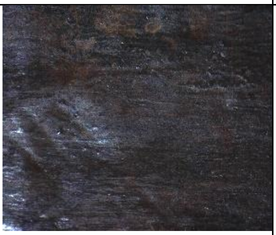	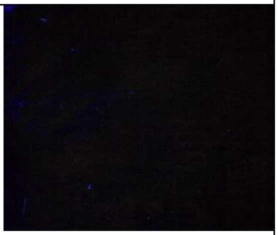
After cleaning	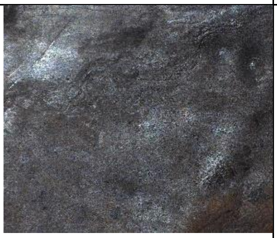	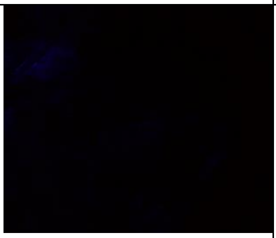	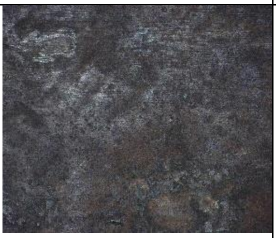	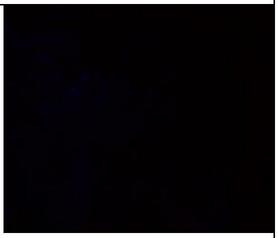
Before cleaning Gel 2	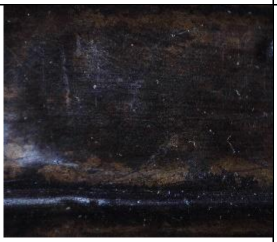	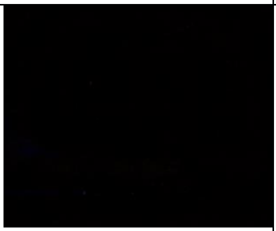	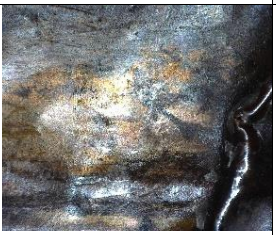	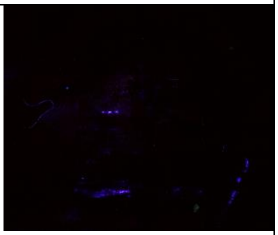
After cleaning	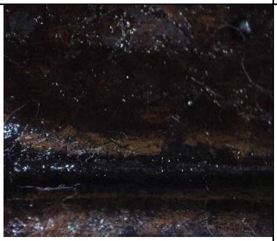	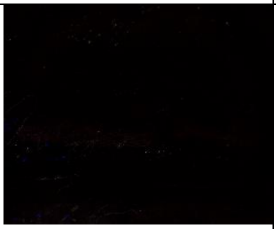	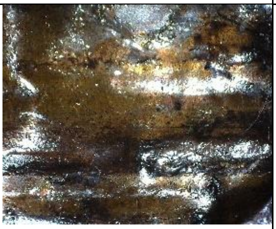	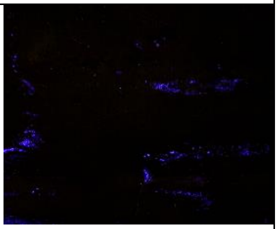

gels

MDPI

Before cleaning<br>Gel 3	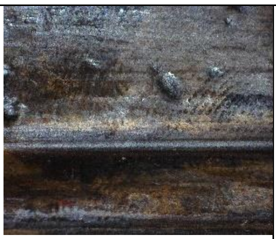	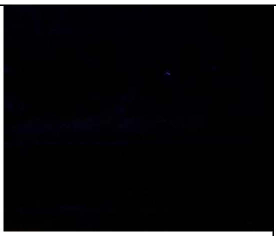	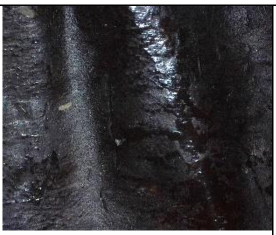	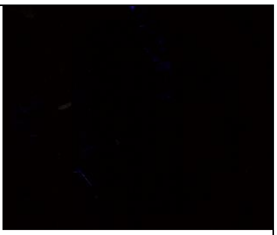
After cleaning	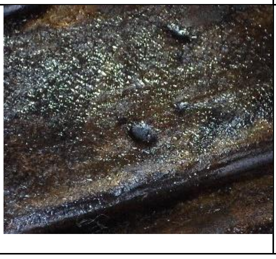	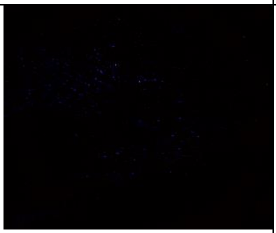	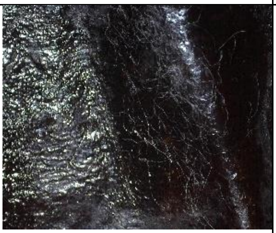	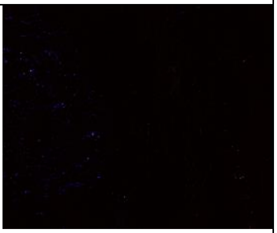
Methyl Myristate (MM)				
Before cleaning	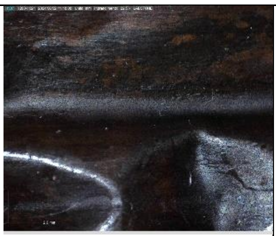	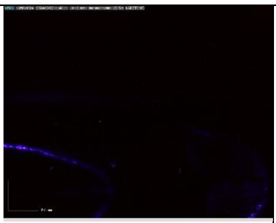	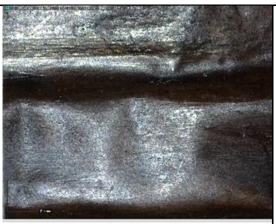	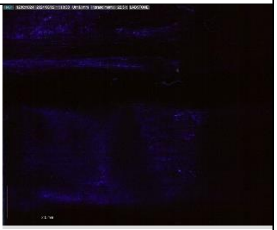
After cleaning	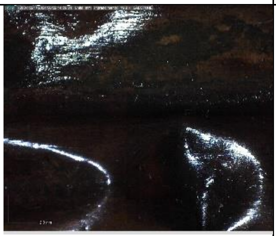	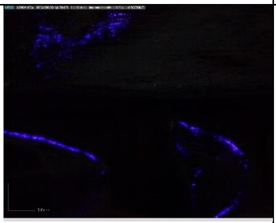	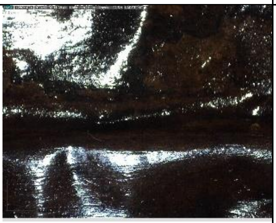	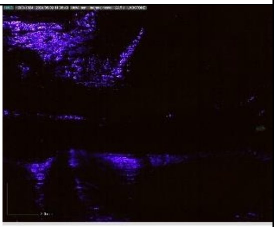
Before cleaning<br>Gel 4	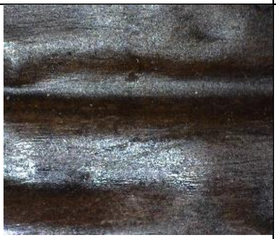	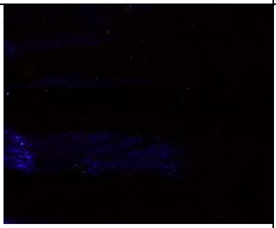	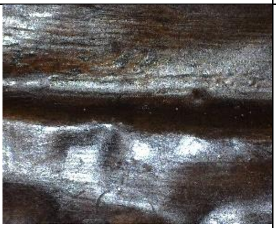	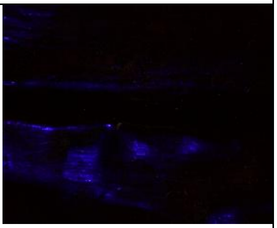
After cleaning	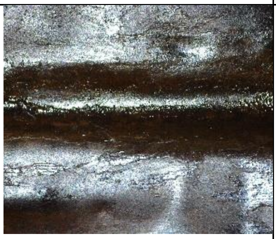	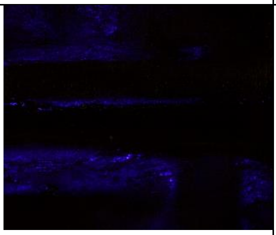	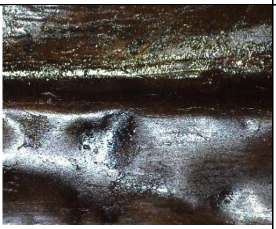	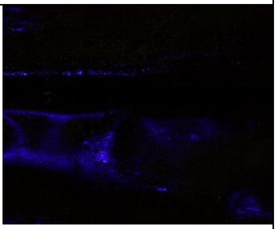
Before cleaning<br>Gel 5	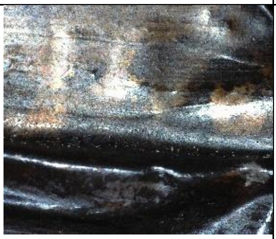	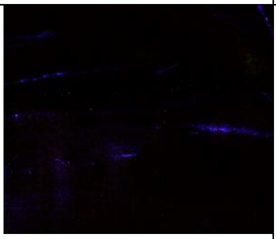	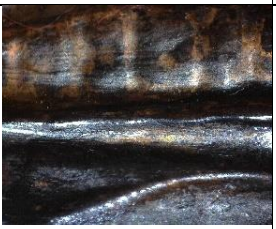	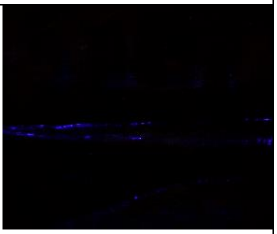

After cleaning	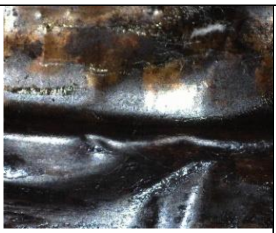	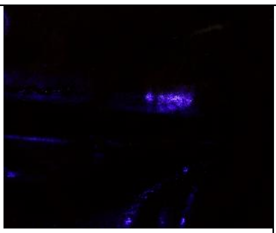	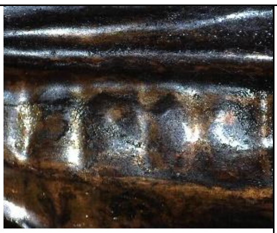	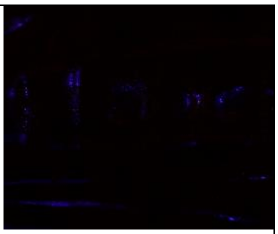
Before cleaning<br>Gel 6	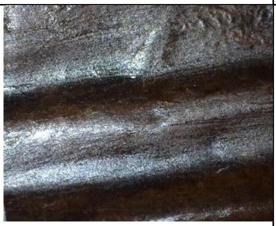	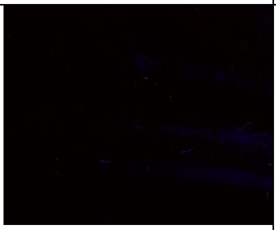	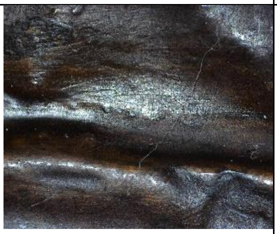	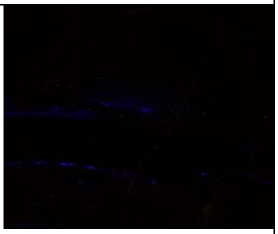
After cleaning	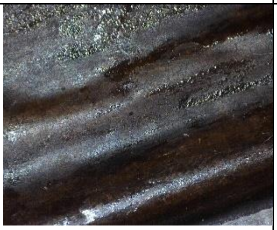	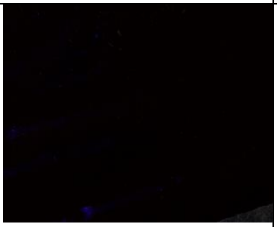	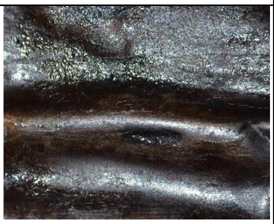	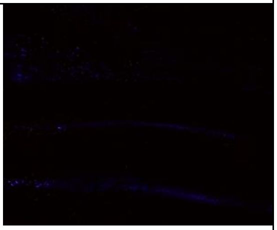
ISOPROPYL PALMITATE (IPP)				
Before cleaning	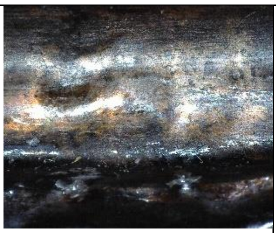	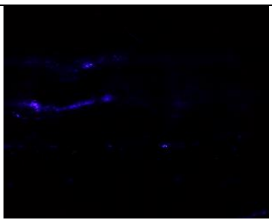	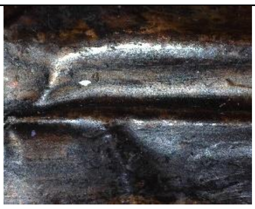	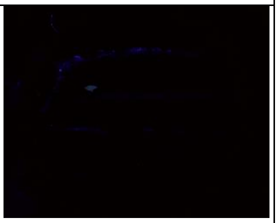
After cleaning	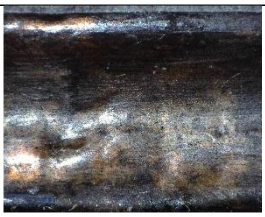	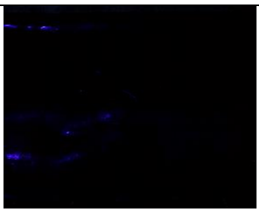	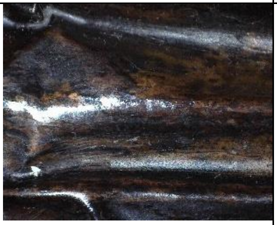	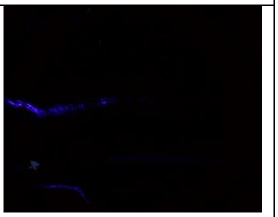
Before cleaning<br>Gel 7	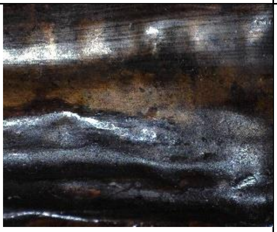	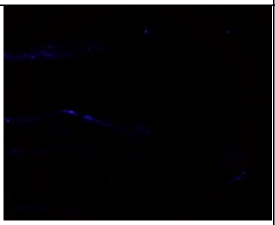	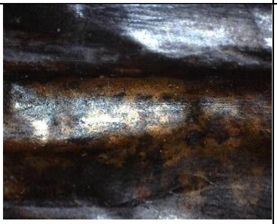	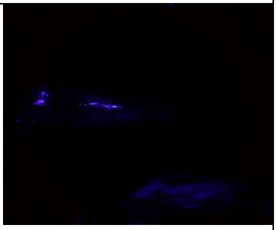
After cleaning	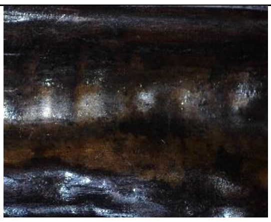	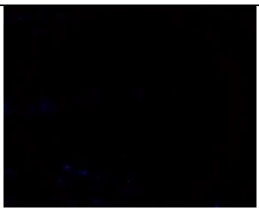	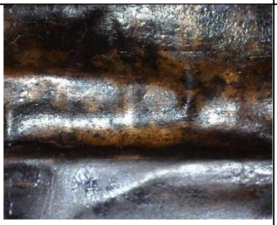	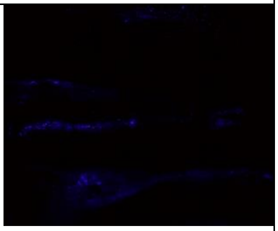

Before cleaning Gel 8	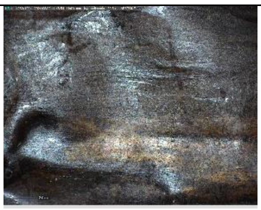	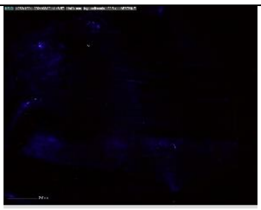	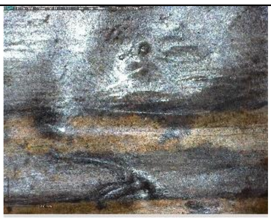	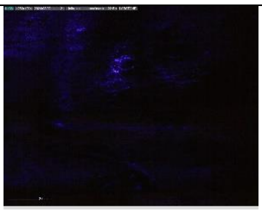
After cleaning	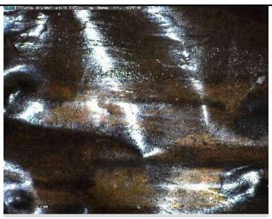	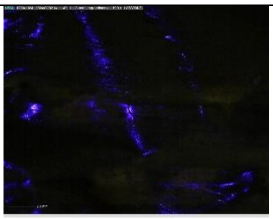	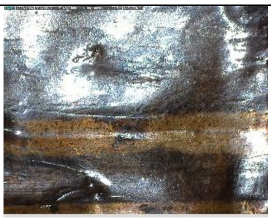	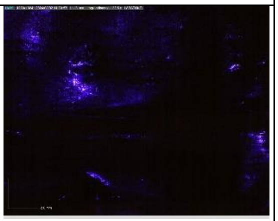
Before cleaning Gel 9	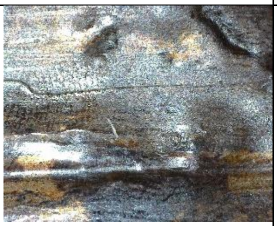	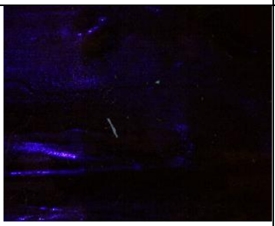	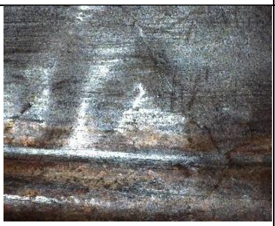	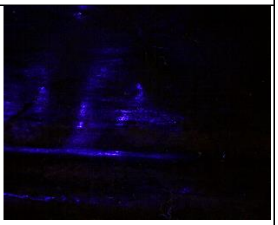
After cleaning	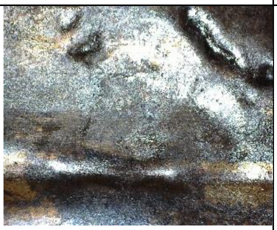	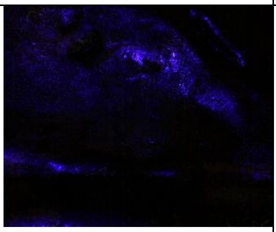	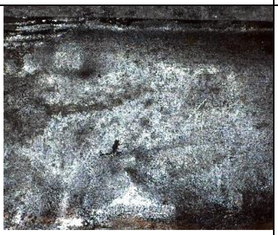	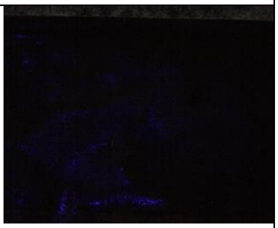

Table S6. Observation under digital light microscopy on the frame area treated with Zapon protective. Cleaning treatment using Ligroin, Methyl Myristate and Isopropyl Palmitate gels.

ZAPON	Aged		Not Aged	
LIGROIN				
Before cleaning	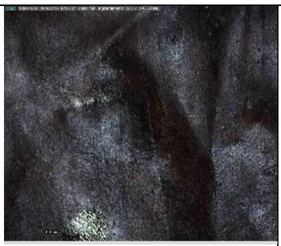	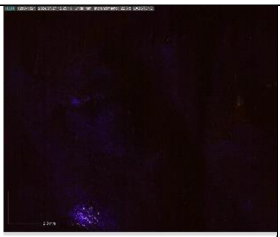	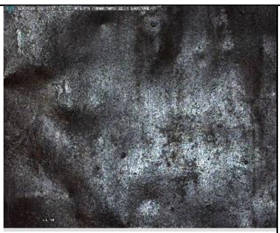	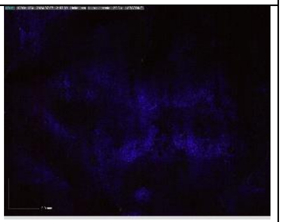
After cleaning	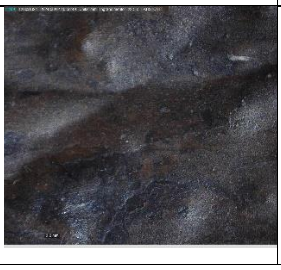	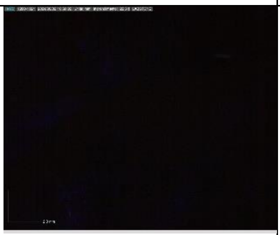	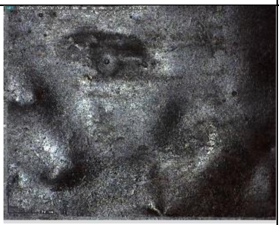	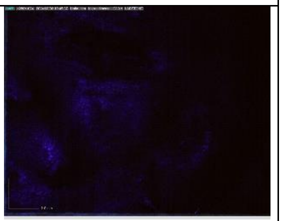

gels

Before cleaning<br>Gel 1	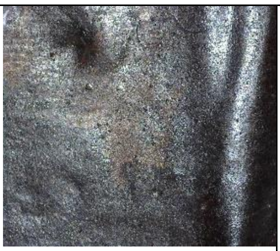	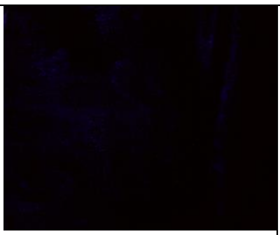	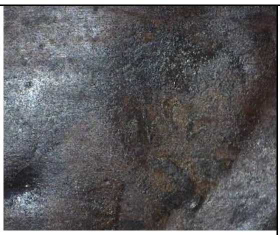	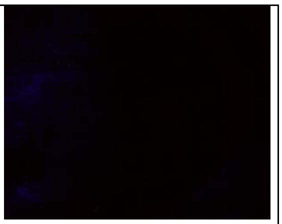
After cleaning	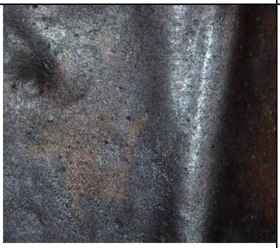	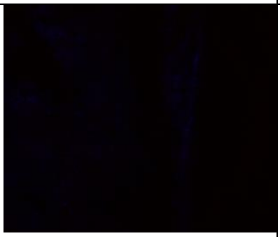	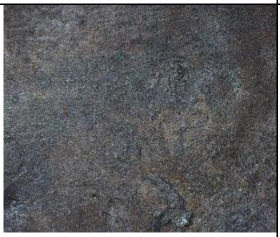	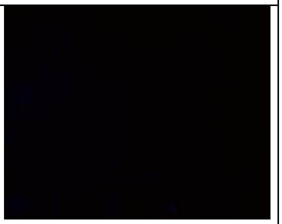
Before cleaning<br>Gel 2	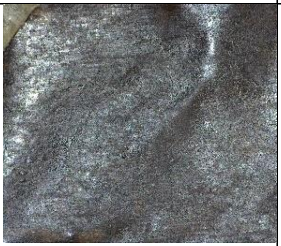	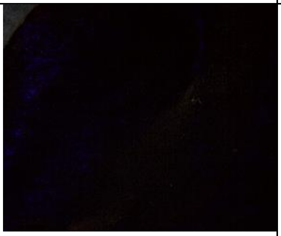	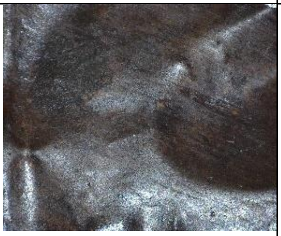	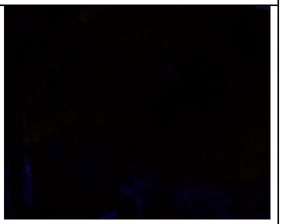
After cleaning	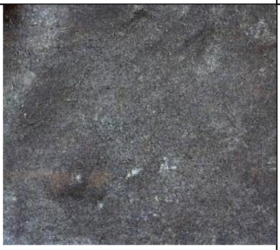	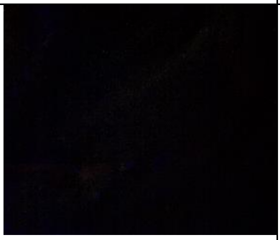	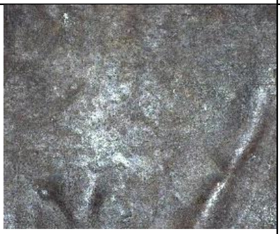	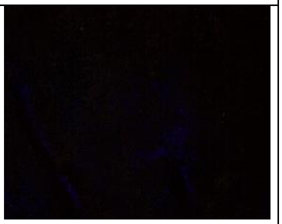
Before cleaning<br>Gel 3	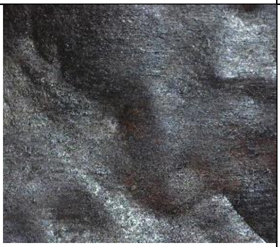	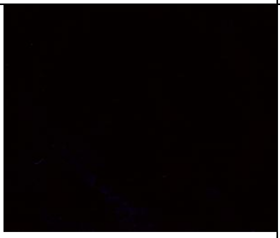	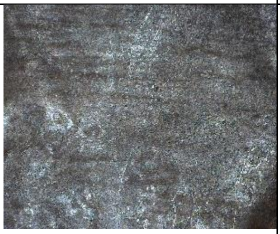	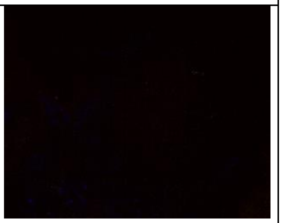
After cleaning	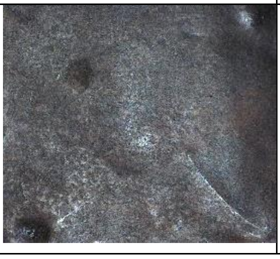	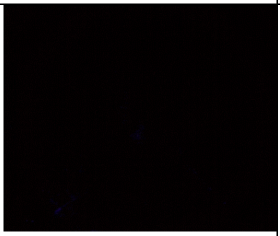	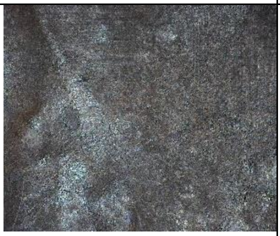	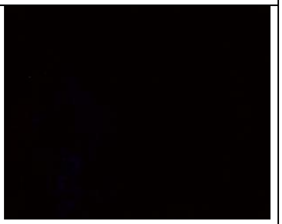
Methyl Myristate				
Before cleaning	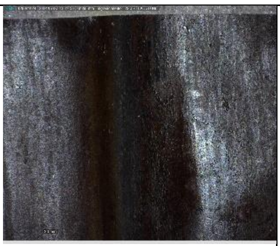	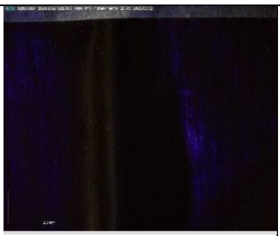	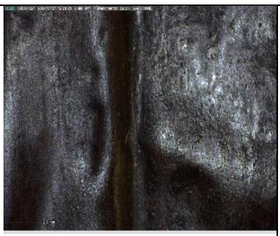	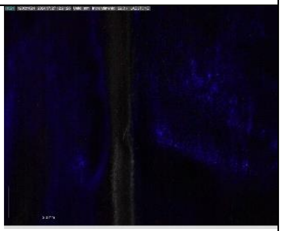

After cleaning	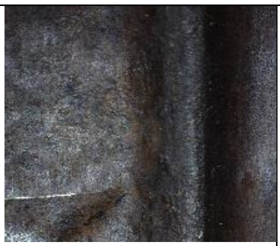	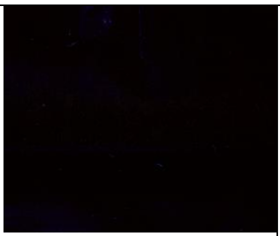	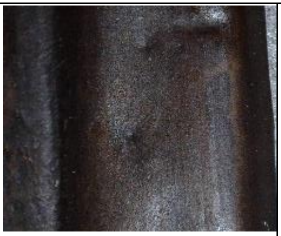	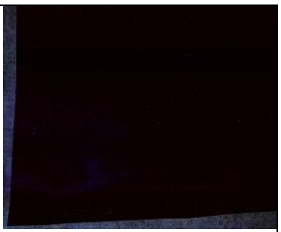
Before cleaning Gel 4	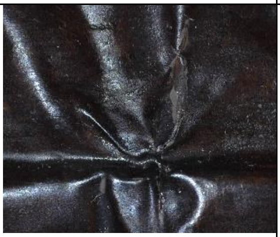	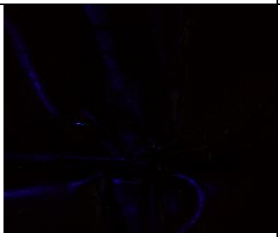	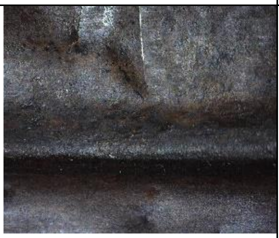	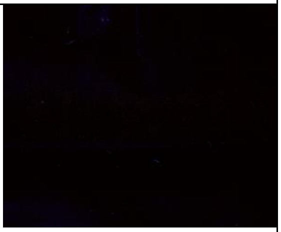
After cleaning	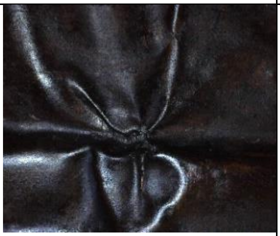	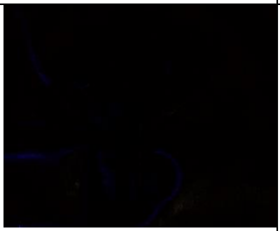	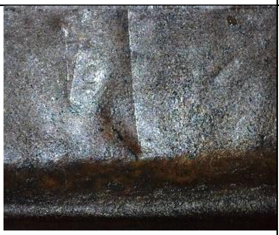	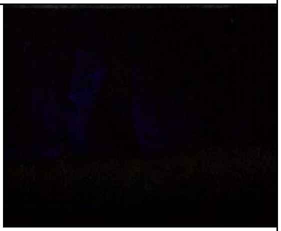
Before cleaning Gel 5	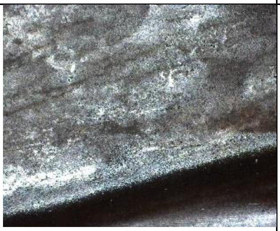	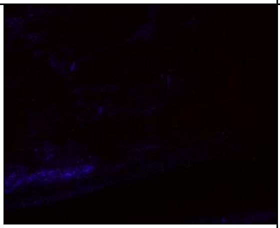	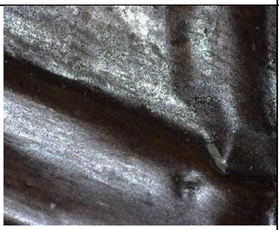	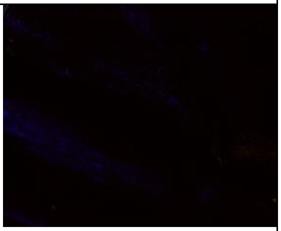
After cleaning	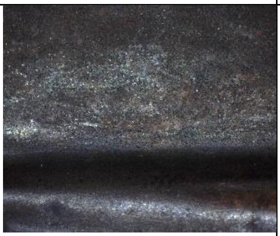	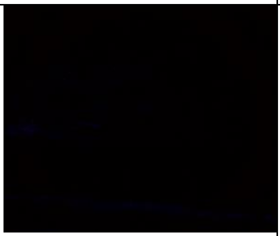	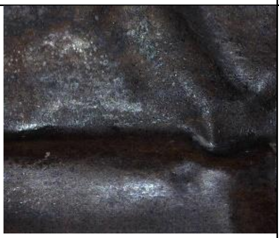	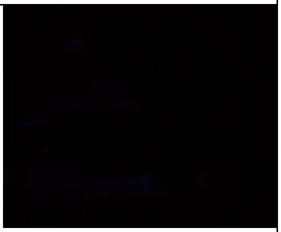
Before cleaning Gel 6	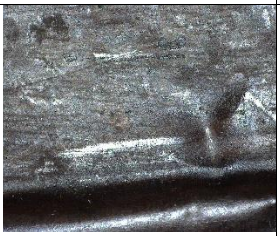	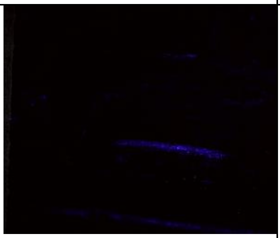	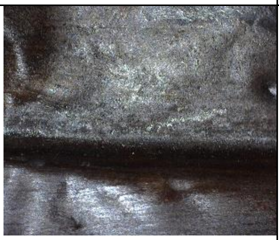	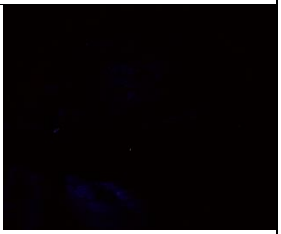
After cleaning	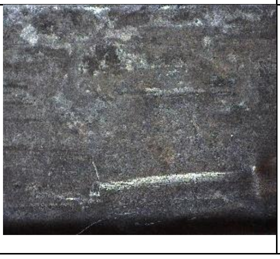	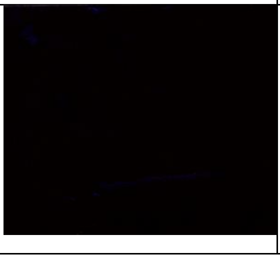	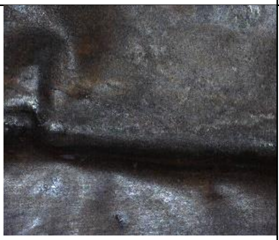	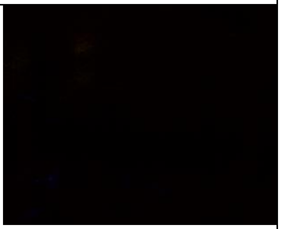
ISOPROPYL PALMITATE (IPP)				

Before cleaning	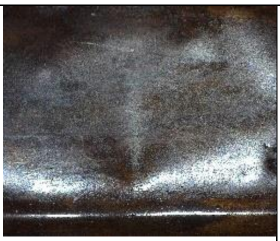	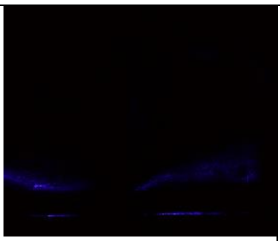	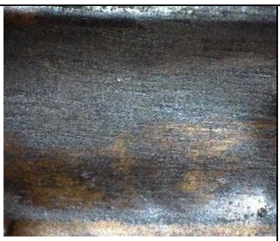	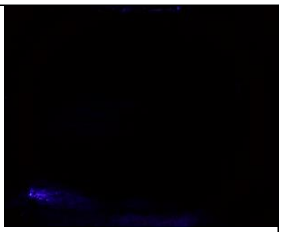
After cleaning	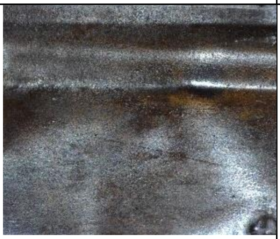	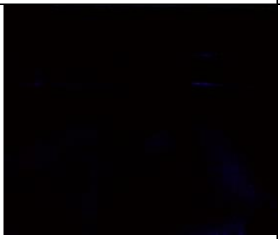	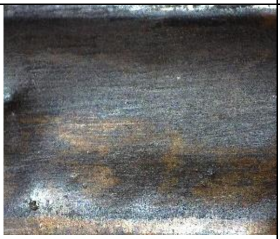	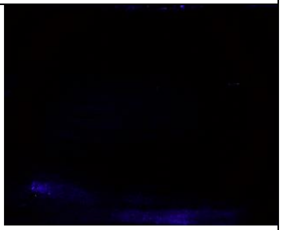
Before cleaning<br>Gel 7	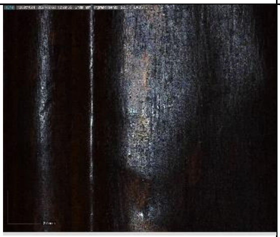	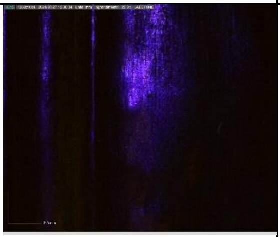	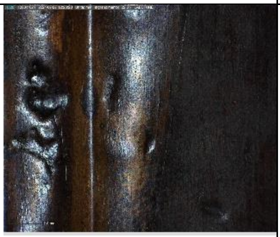	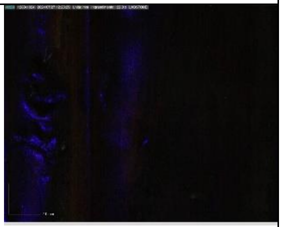
After cleaning	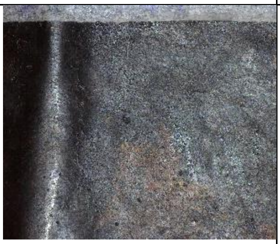	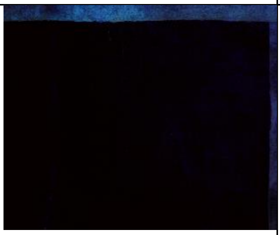	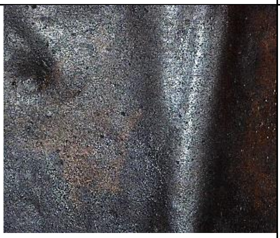	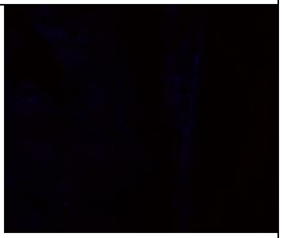
Before cleaning<br>Gel 8	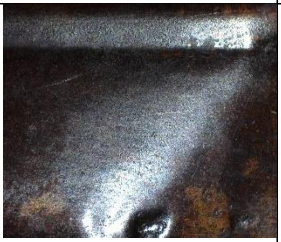	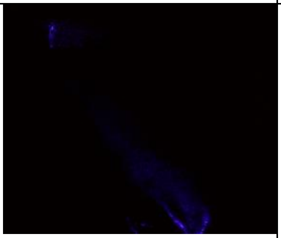	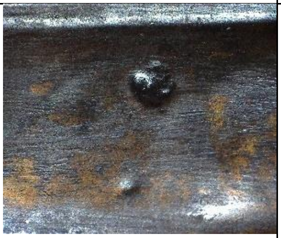	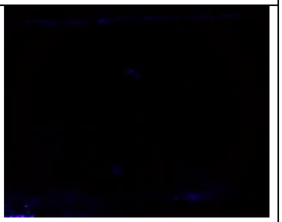
After cleaning	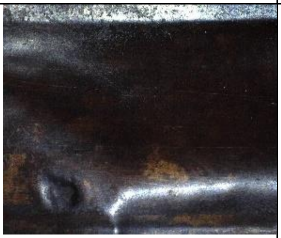	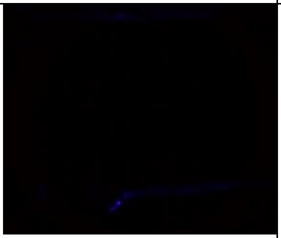	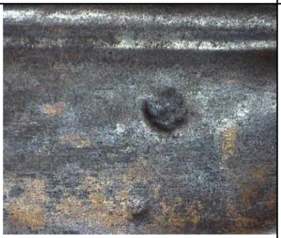	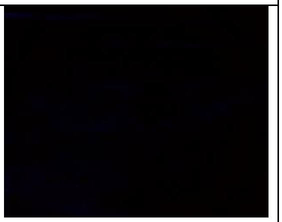
Before cleaning<br>Gel 9	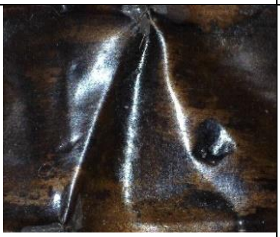	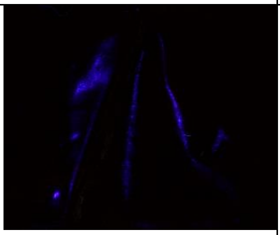	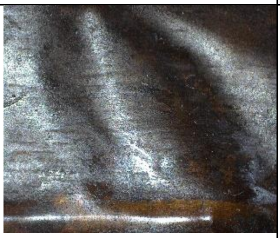	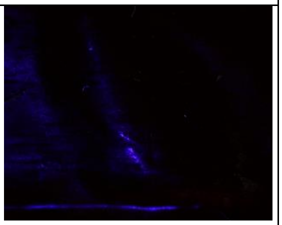

After cleaning	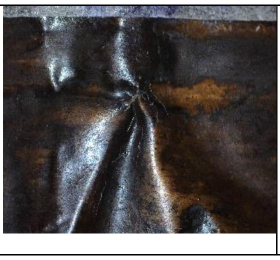	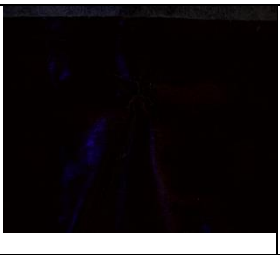	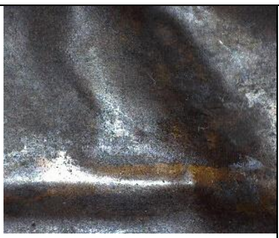	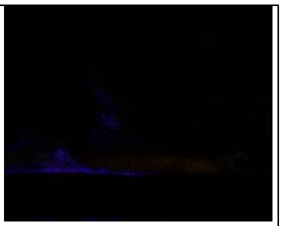

Table S7. Observation under digital light microscopy on the frame area treated with Incral + Soter multilayer. Cleaning treatment using Ligroin, Methyl Myristate and Isopropyl Palmitate gels.				
INCRAL + SOTER	Aged		Not Aged	
LIGROIN				
Before cleaning	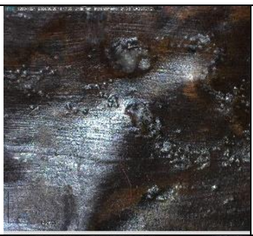	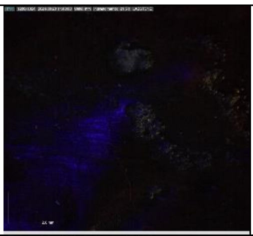	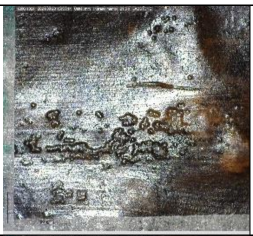	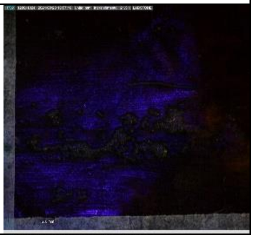
After cleaning	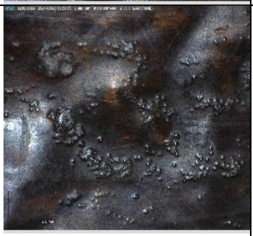	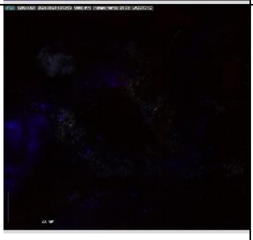	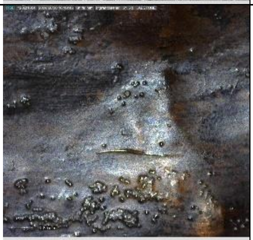	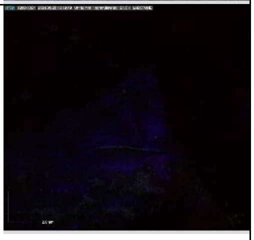
Before cleaning Gel 1	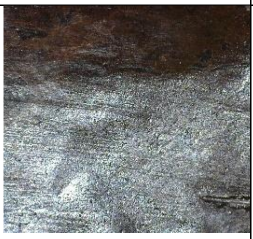	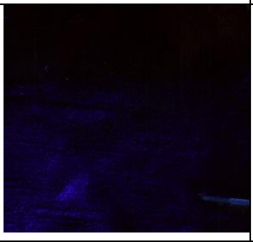	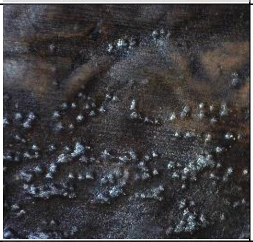	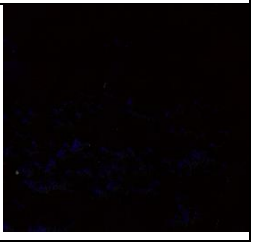
After cleaning	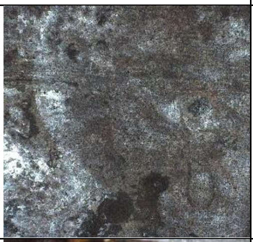	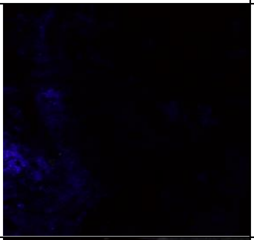	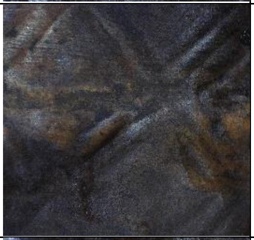	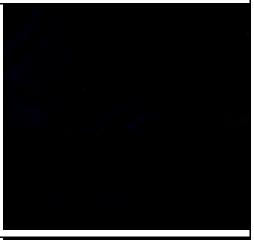
Before cleaning Gel 2	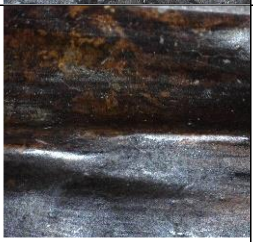	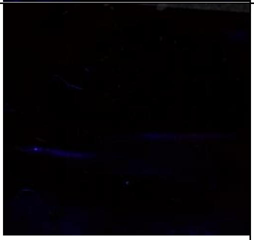	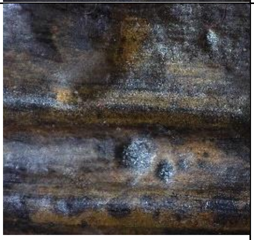	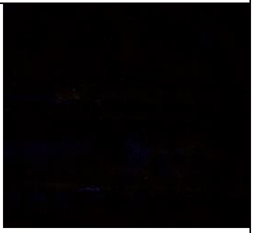

After cleaning	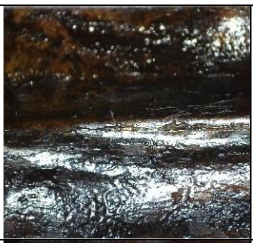	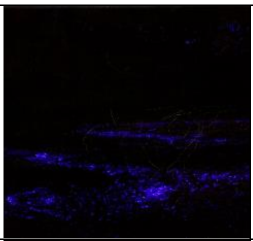	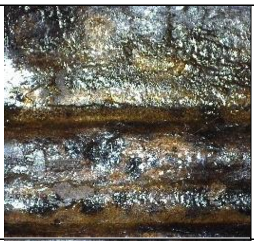	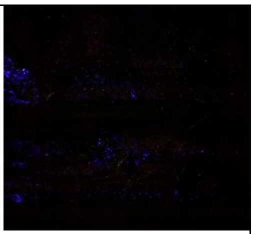
Before cleaning<br>Gel 3	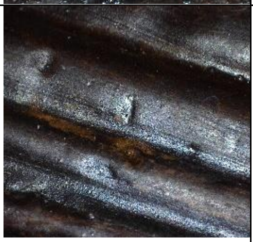	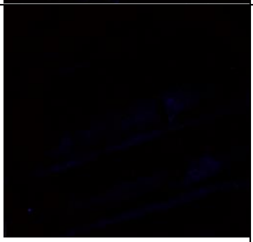	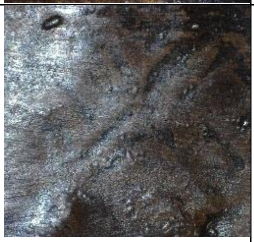	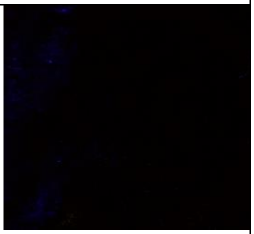
After cleaning	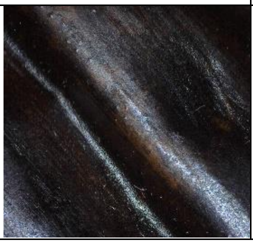	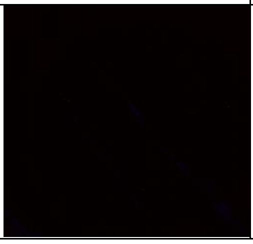	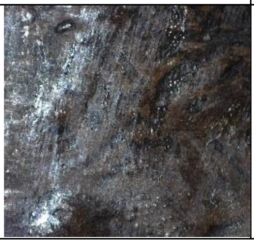	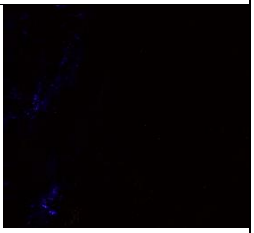
Methyl Myristate (MM)				
Before cleaning	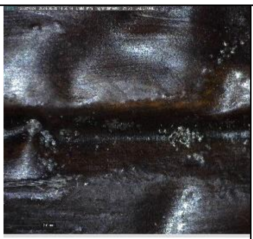	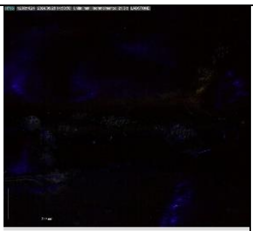	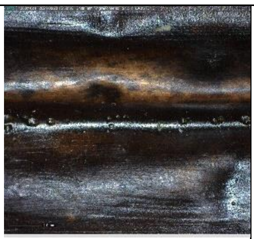	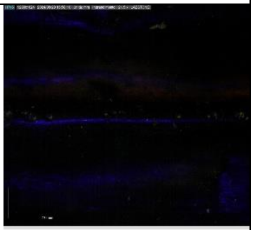
After cleaning	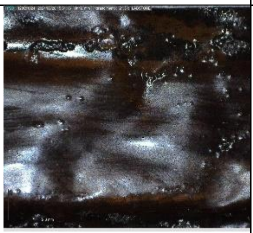	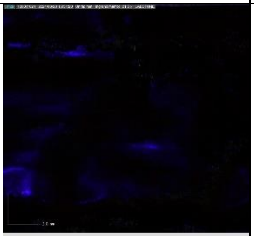	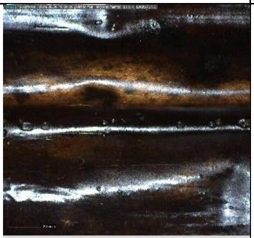	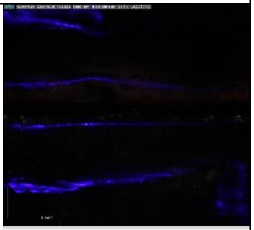
Before cleaning<br>Gel 4	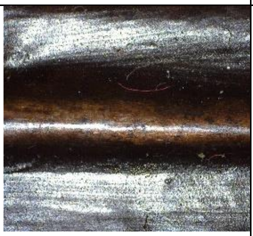	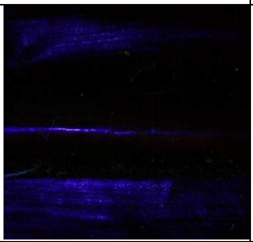	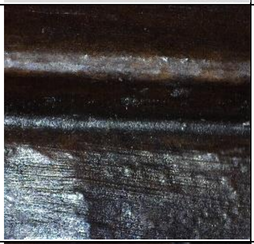	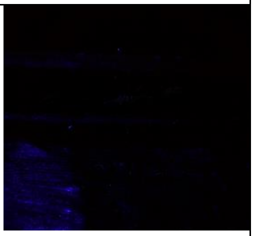
After cleaning	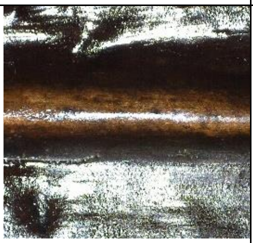	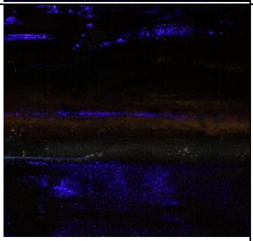	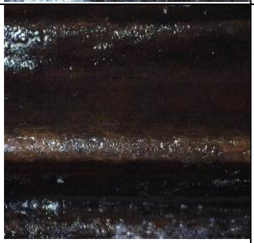	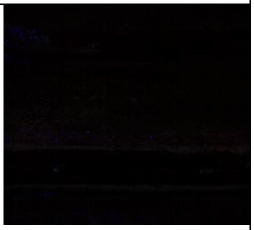

Before cleaning Gel 5	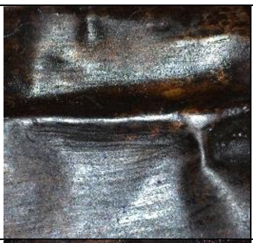	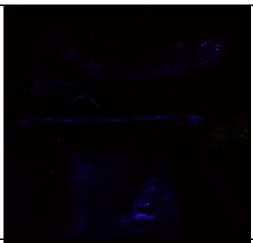	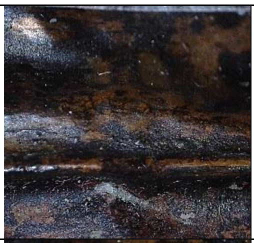	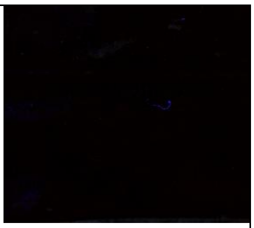
After cleaning	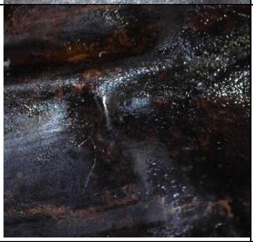	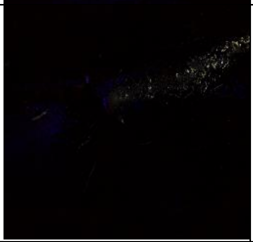	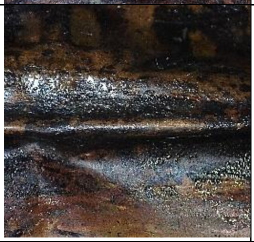	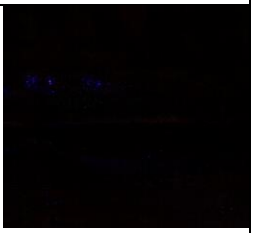
Before cleaning Gel 6	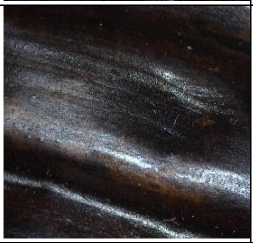	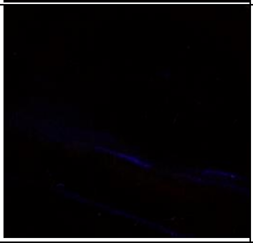	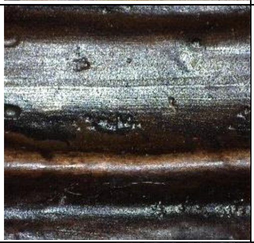	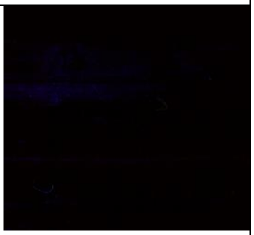
After cleaning	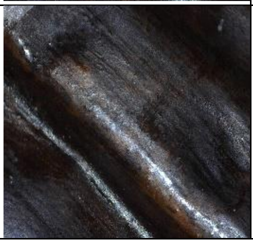	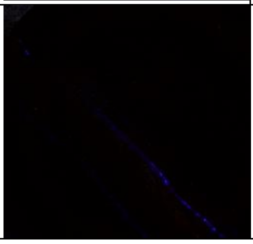	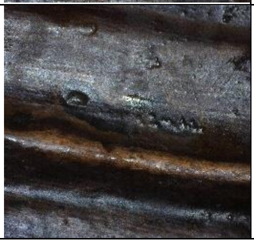	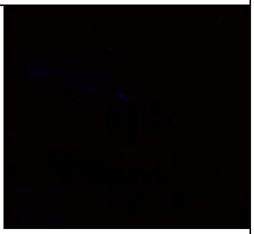
ISOPROPYL PALMITATE (IPP)				
Before cleaning	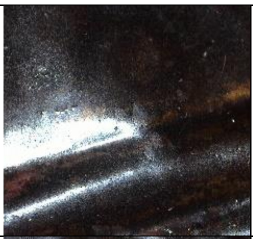	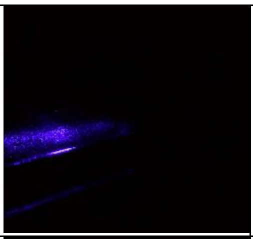	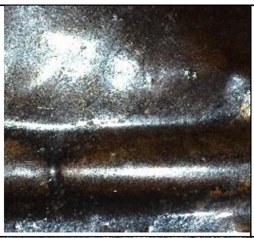	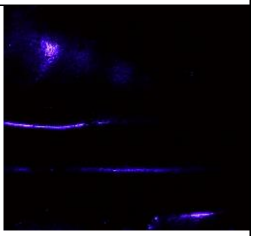
After cleaning	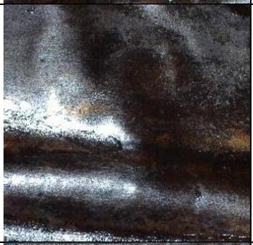	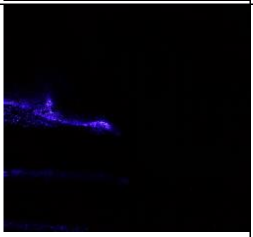	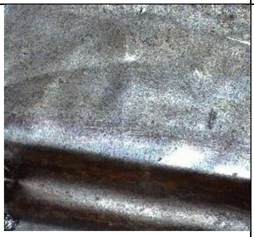	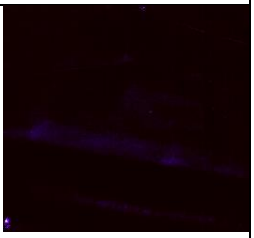
Before cleaning Gel 7	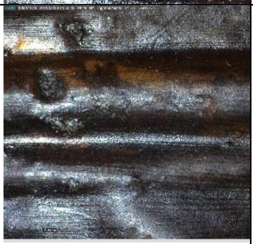	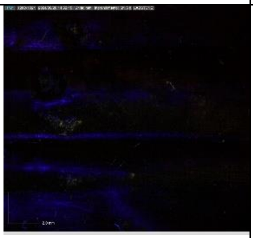	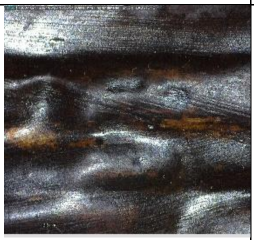	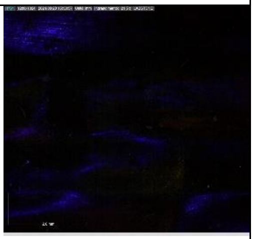

After cleaning	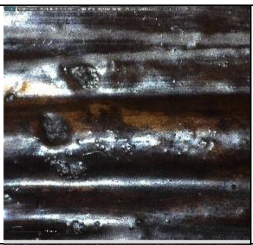	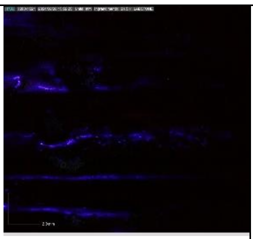	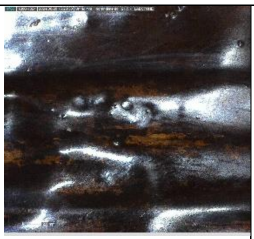	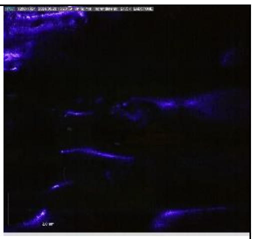
Before cleaning Gel 8	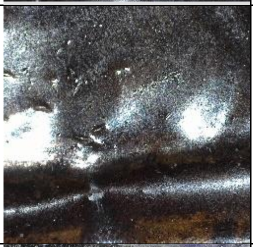	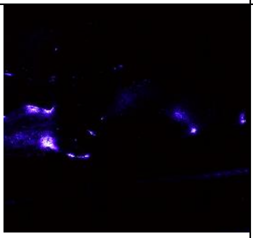	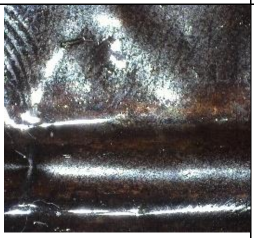	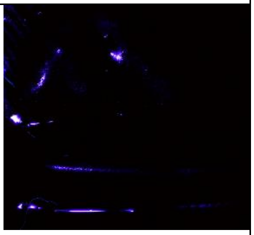
After cleaning	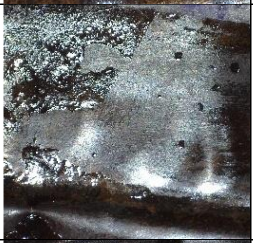	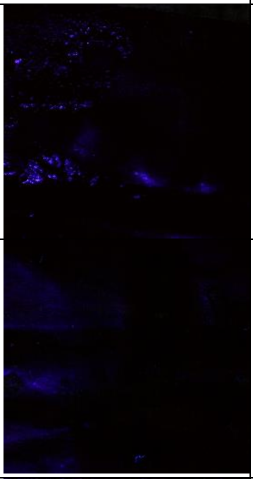	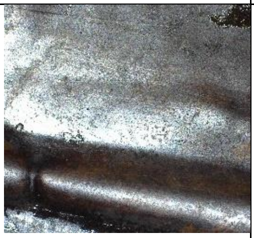	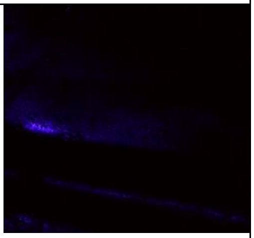
Before cleaning Gel 9	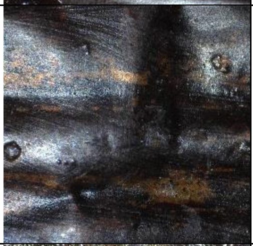		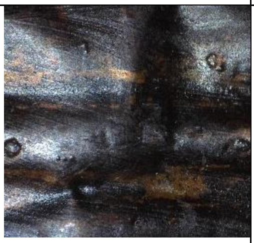	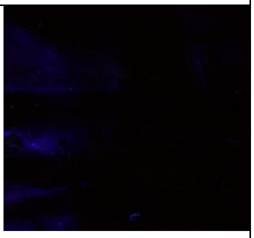
After cleaning	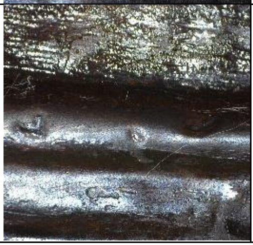	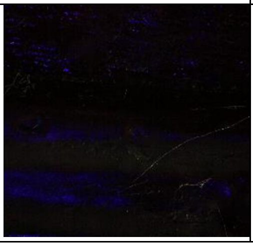	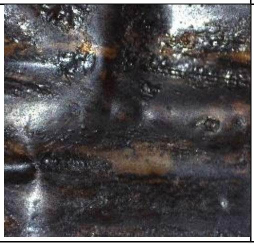	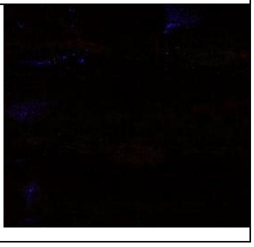

Table S8. Observation under digital light microscopy on the frame area treated with Soter's protective agent. Cleaning treatment using Ligroin, Methyl Myristate and Isopropyl Palmitate gels.

SOTER	Aged		Not Aged	
	LIGROIN			
Before cleaning	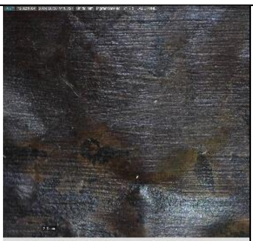	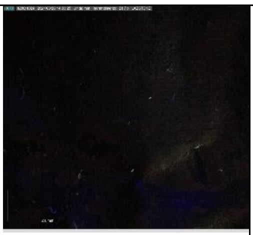	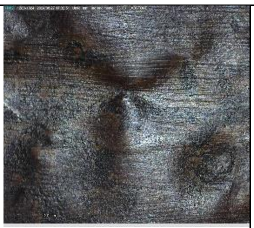	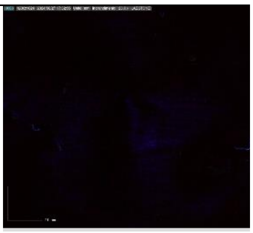
After cleaning	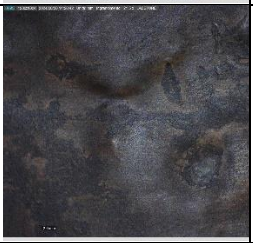	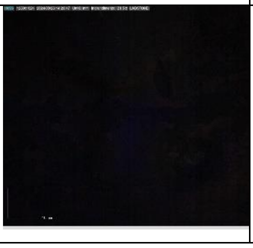	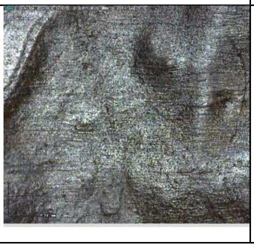	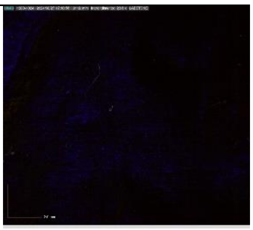

Before cleaning Gel 1	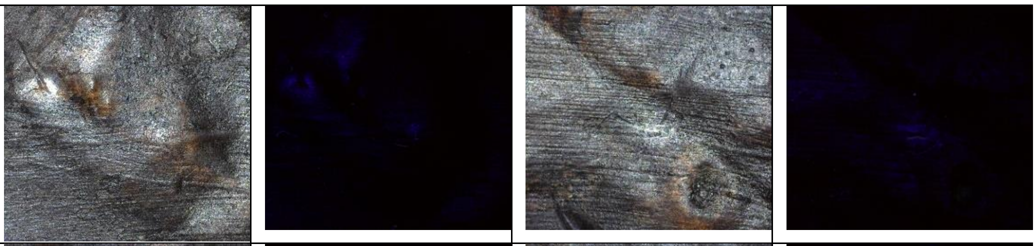
After cleaning	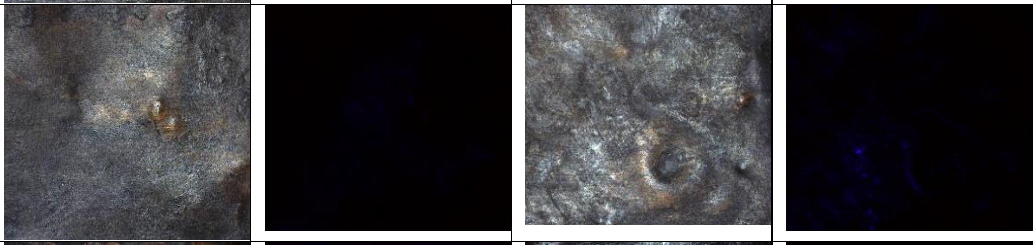
Before cleaning Gel 2	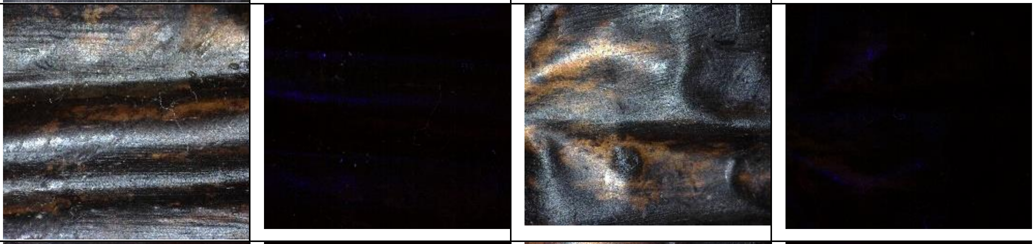
After cleaning	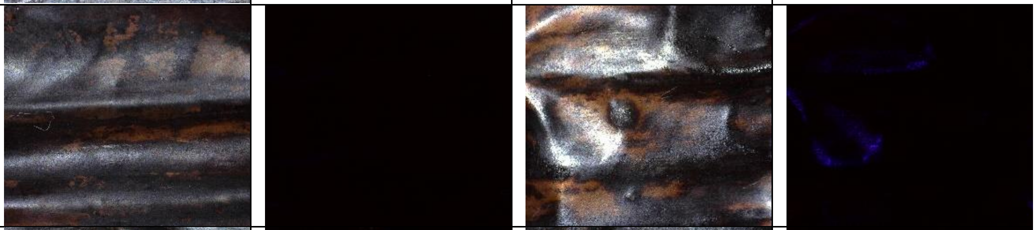
Before cleaning Gel 3	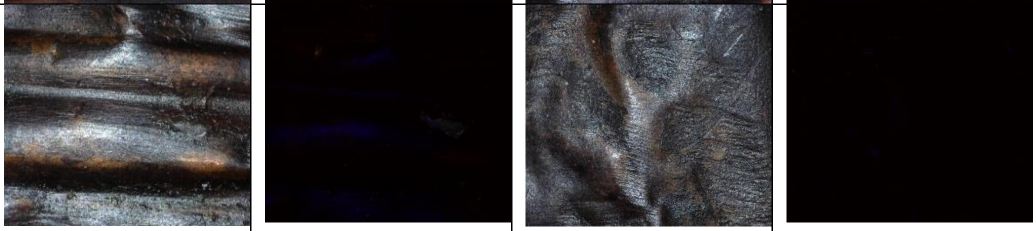
After cleaning	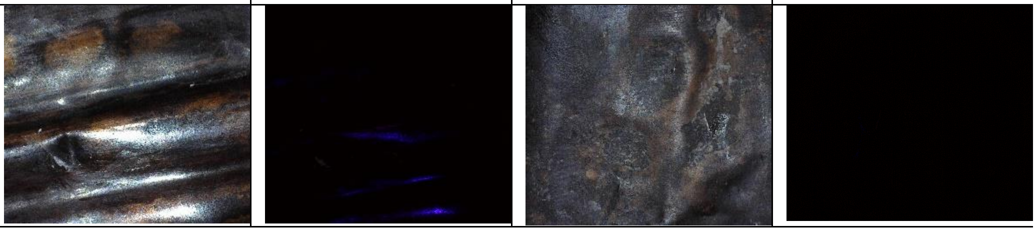
Methyl Myristate (MM)	
Before cleaning	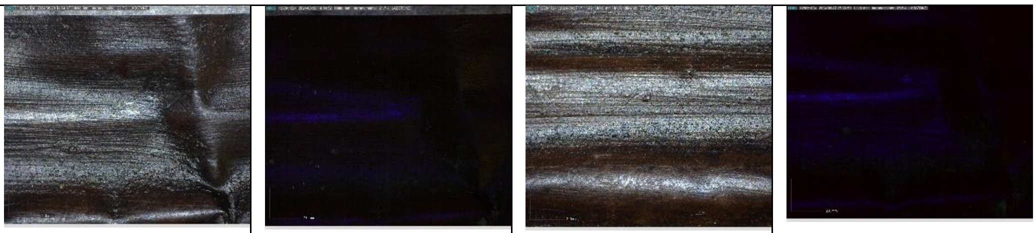

After cleaning	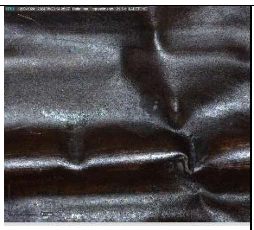	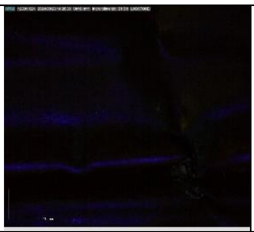	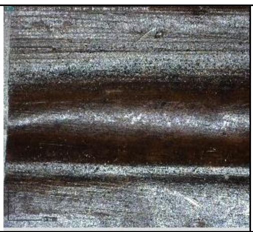	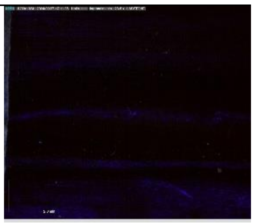
Before cleaning<br>Gel 4	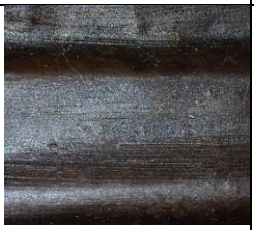	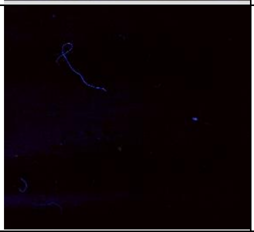	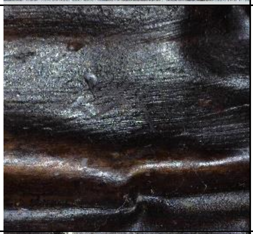	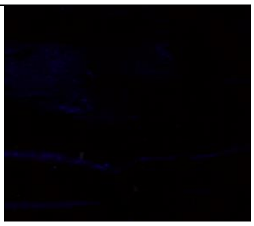
After cleaning	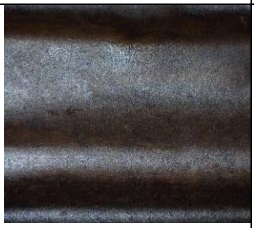	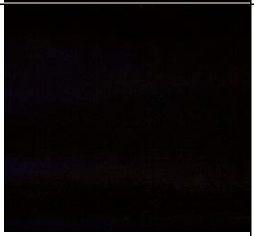	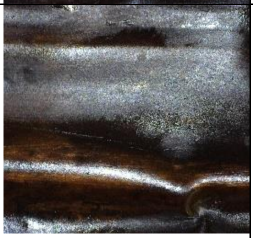	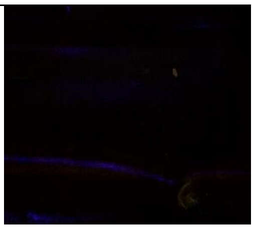
Before cleaning<br>Gel 5	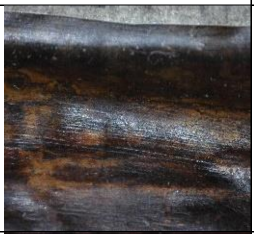	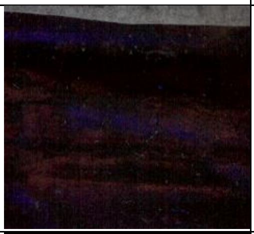	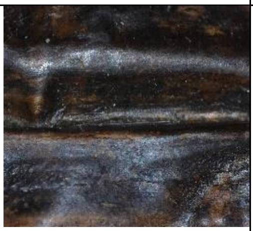	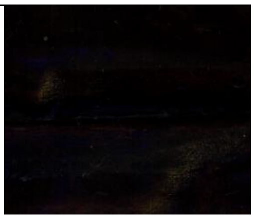
After cleaning	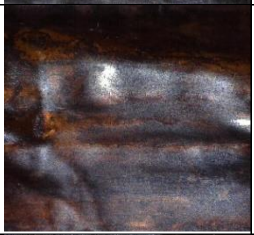	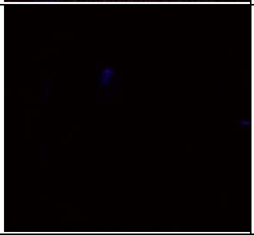	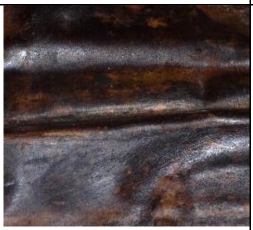	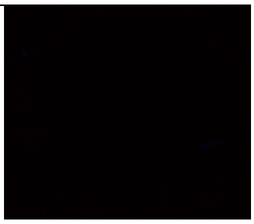
Before cleaning<br>Gel 6	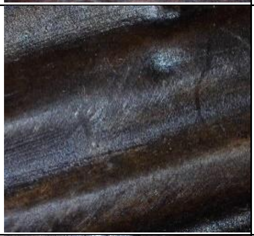	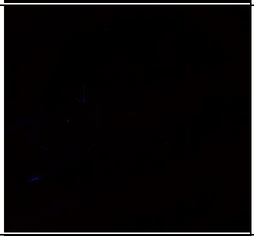	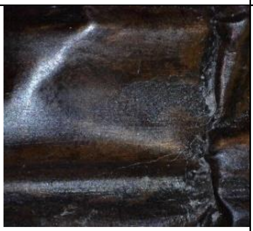	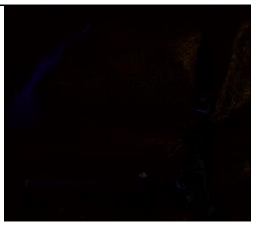
After cleaning	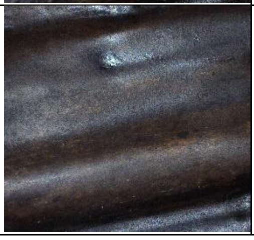	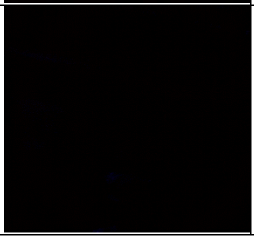	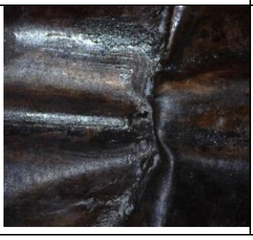	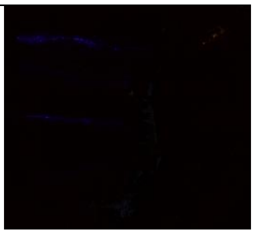
ISOPROPIL PALMITATE (IPP)				

gels

Before cleaning	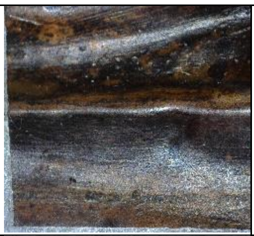	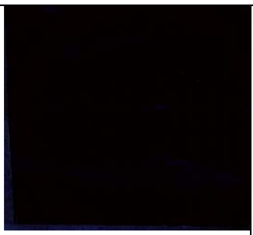	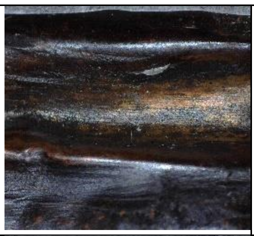	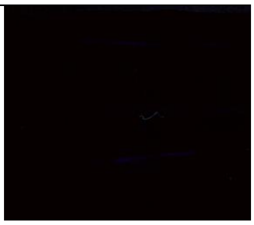
After cleaning	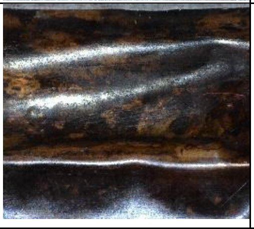	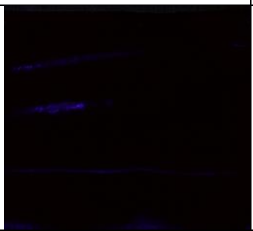	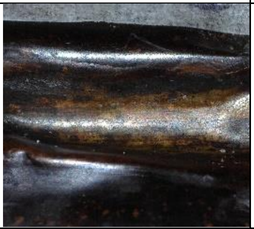	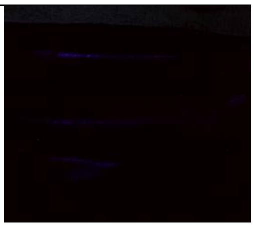
Before cleaning Gel 7	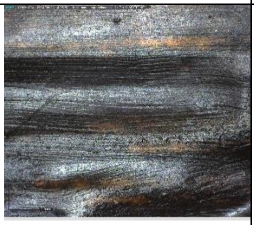	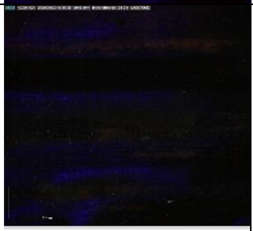	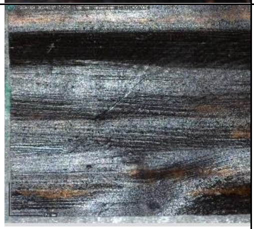	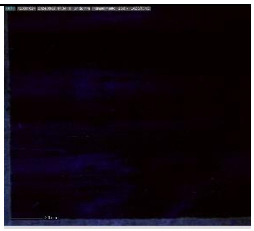
After cleaning	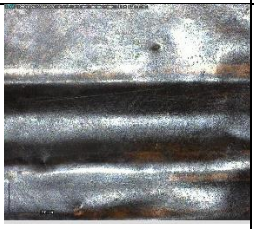	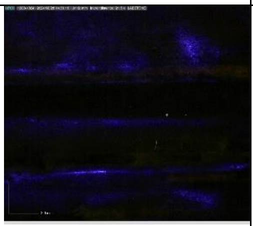	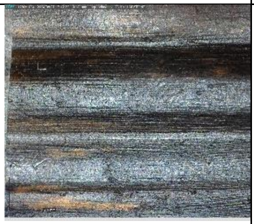	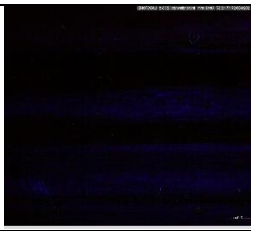
Before cleaning Gel 8	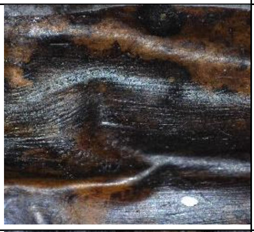	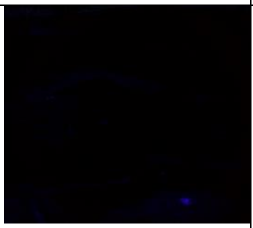	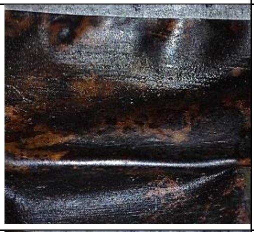	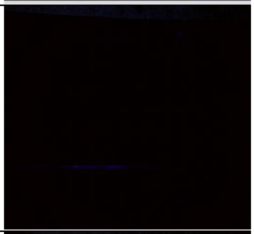
After cleaning	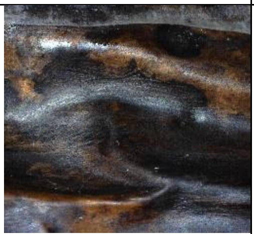	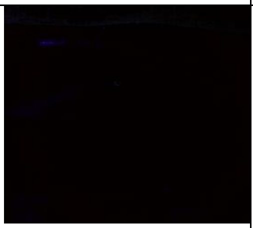	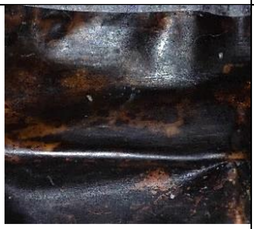	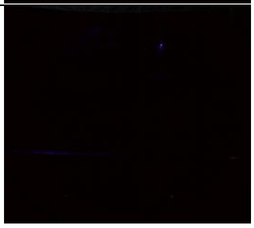
Before cleaning Gel 9	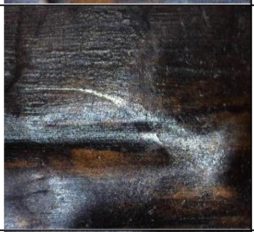	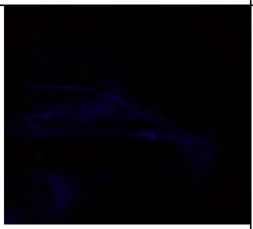	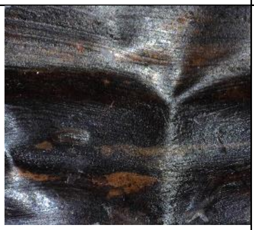	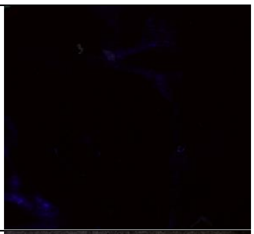
After cleaning	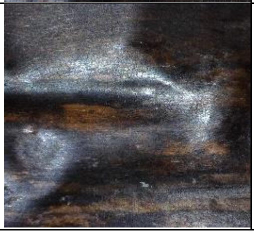	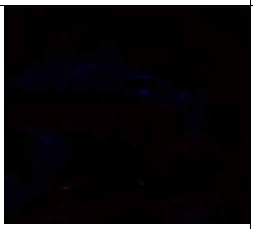	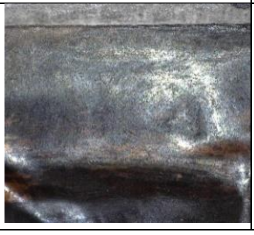	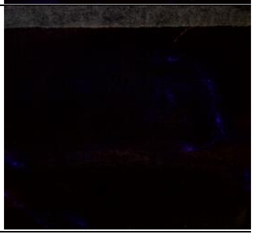
